# Gibberellin signaling mediates lateral root inhibition in response to K^+^-deprivation

**DOI:** 10.1093/plphys/kiaa093

**Published:** 2021-01-06

**Authors:** Flora M Hetherington, Medhavi Kakkar, Jennifer F Topping, Keith Lindsey

**Affiliations:** Department of Biosciences, Durham University, Durham DH1 3LE, UK

## Abstract

The potassium ion (K^+^) is vital for plant growth and development, and K^+^-deprivation leads to reduced crop yields. Here we describe phenotypic, transcriptomic, and mutant analyses to investigate the signaling mechanisms mediating root architectural changes in Arabidopsis (*Arabidopsis thaliana*) Columbia. We showed effects on root architecture are mediated through a reduction in cell division in the lateral root (LR) meristems, the rate of LR initiation is reduced but LR density is unaffected, and primary root growth is reduced only slightly. This was primarily regulated through gibberellic acid (GA) signaling, which leads to the accumulation of growth-inhibitory DELLA proteins. The short LR phenotype was rescued by exogenous application of GA but not of auxin or by the inhibition of ethylene signaling. RNA-seq analysis showed upregulation by K^+^-deprivation of the transcription factors JUNGBRUNNEN1 (JUB1) and the C-repeat-binding factor (CBF)/dehydration-responsive element-binding factor 1 regulon, which are known to regulate GA signaling and levels that regulate DELLAs. Transgenic overexpression of JUB1 and CBF1 enhanced responses to K^+^ stress. Attenuation of the reduced LR growth response occurred in mutants of the CBF1 target gene *SFR6*, implicating a role for JUB1, CBF1, and SFR6 in the regulation of LR growth in response to K^+^-deprivation via DELLAs. We propose this represents a mechanism to limit horizontal root growth in conditions where K^+^ is available deeper in the soil.

## Teaser

The gibberellin pathway is essential for the root response to low K stress in Arabidopsis.

## Introduction

Potassium (K^+^) is one of the most important nutrients that plants need to survive. It is the most abundant cation in land plants, making up 2%–10% of the dry weight of a plant (Leigh and Jones, 1984). It is essential for many functions in the plant, including enzyme activation, stomatal activity, photosynthesis, protein synthesis, and the transport of sugars, water, and nutrients (Prajapati and Modi, 2012). A key role for sufficient K^+^ nutrition has also been identified in plant resistance to various abiotic and biotic stresses (Wang et al., 2013). Despite being one of the most abundant elements on Earth, its availability to plants is often limited. Typically, the concentration of K^+^ in soil is between 0.1 and 6 mM (Adams, 1971) although acidic soils and intensive farming can lead to its depletion. To alleviate this problem, it is necessary to apply large amounts of K^+^ fertilizer to soils. K^+^-deprivation is a problem particularly in developing countries, as the addition of K^+^ fertilizers is often neglected or not possible for economic reasons.

K^+^-deprivation has an adverse impact on plant growth, both of the above-ground organs as well as the roots (Chérel et al., 2014). Work over many years in Arabidopsis (*Arabidopsis thaliana*) shows a range of nutrient deficiencies (Gruber et al., 2013), including K^+^-deprivation, leads to inhibition of lateral root (LR) initiation and development (Armengaud et al., 2004; Shin and Schachtman, 2004), an increase in root hair elongation (Desbrosses et al., 2003; López-Bucio et al., 2003; Jung et al., 2009), inhibition of growth of the primary root (PR; Jung et al., 2009; Kim et al., 2010), and a mild agravitropic response (Vicente-Agullo et al., 2004). Kellermeier et al. (2013) reported a phenotypic gradient of growth responses to K^+^-deprivation in different accessions of Arabidopsis, explaining conflicting results published previously. This phenotypic gradient has two extremes based on the trade-off between the growth of the PR and LRs. These authors defined two strategies: the first strategy results in the maintenance of the growth of the PR as K^+^ decreases but restricts the growth of the LRs, whereas the second strategy restricts the growth of the PR in favor of elongation of LRs (Kellermeier et al., 2013). Genetic variation in CLASSY1 (CLSY1), a component of the RNA-directed DNA methylation mechanism, in part accounts for phenotypic response changes via effects on the expression of IAA27, a negative regulator of LR development (Shahzad et al., 2020). This suggests a role for auxin in LR development in response to low K^+^, but auxin acts in a network with other signaling pathways (Moore et al. 2015), and understanding this relationship requires elucidation.

Much of the work surrounding K^+^-deprivation has focused on the changes in K^+^ transporter levels and uptake kinetics and the hormonal signaling pathways involved in their regulation (Jung et al., 2009; Nam et al., 2012). However, much less is known about the hormonal control of the root architectural changes displayed in response to K^+^-deprivation. Ethylene and reactive oxygen species (ROS) have been linked to increased elongation of root hairs (Pitts et al., 1998; Jung et al., 2009) and to reduction in PR growth (Jung et al., 2009) in response to K^+^-deprivation, and there is mounting evidence to suggest that auxin also plays a key role in the response to K^+^-deprivation (Armengaud et al., 2004; Vicente-Agullo et al., 2004; Shin et al., 2007; Rigas et al., 2013; Shahzad et al., 2020). Analysis of cytokinin synthesis mutants has also indicated a role for cytokinin in the response to K^+^-deprivation, as the mutants display an enhanced K^+^-deprivation response (Nam et al., 2012). A reversible increase in jasmonic acid levels in response to K^+^-deprivation (Armengaud et al., 2004; Cao et al., 2006) as well as an overlap in transcript profiles between K^+^ starvation and the response to pathogen/herbivory have also been used to suggest that defense pathways are upregulated during K^+^ limiting conditions (Armengaud et al., 2010).

The objective of the work described here was to investigate the mechanistic basis of root architectural responses to K^+^-deprivation in the Arabidopsis accession Columbia (Col-0). We describe the root architectural and global gene expression responses of Col-0 to K^+^-deprivation. This analysis led us to investigate the roles of the hormones auxin, ethylene, and gibberellin (GA) and transcription factor (TF) function in this response. We propose a role for GA regulation of root development under K^+^-deprivation.

## Results

### LR primordia initiate but do not elongate in response to K^+^-deprived conditions

**Figure 1:**
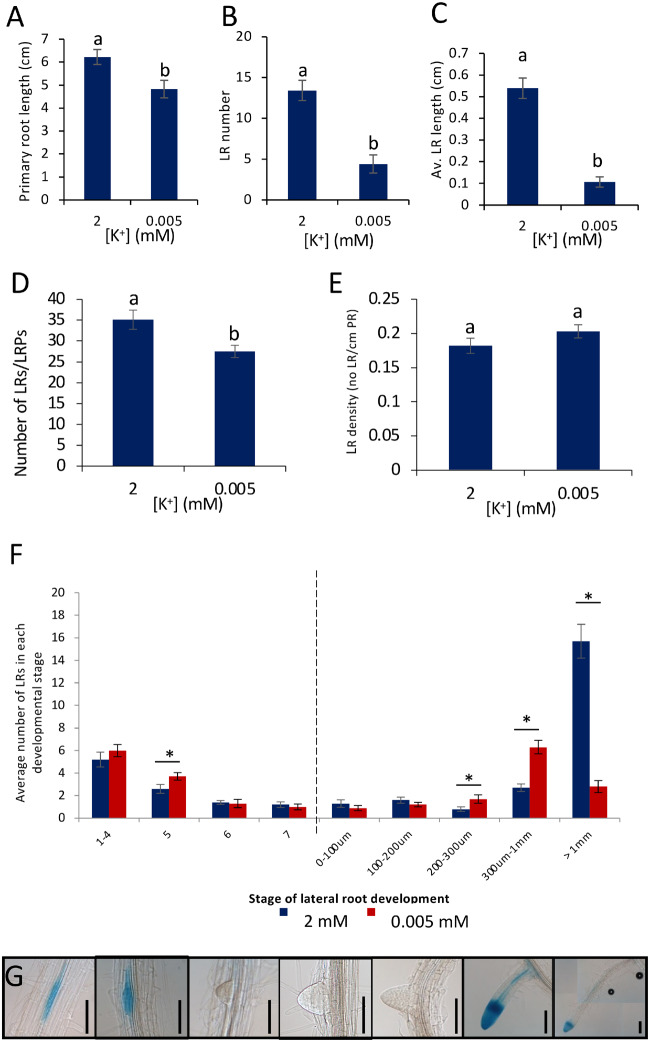
Effect of K^+^-deprivation on root development of Arabidopsis. WT accession Col-0 seedlings were grown for 4 d on half MS10 agar plates followed by 8 d on vertical agar plates supplemented with either 2 mM or 0.005 mM K^+^. A, Average PR length. B, Average emerged LR number. C, Average LR length. D, Average number of LRP and LRs per seedling. E, Average LR density (total LRs/PR length) per seedling. F, Average number of LRs in each stage of development on each PR. Dotted line indicates point of emergence from the PR. G, *DR5::GUS* staining pattern of LR development. Stages shown are (left-right) 1–4, 5, 6, and 7 as defined by Malamy and Benfey (1997), 0–100 *µ*m, 300 µm to 1 mm, and >1 mm. Scale bars = 100 µm. Values represent means ± se, *n* ≥ 10. Letters indicate significance with independent samples *t* test (*P* < 0.05) (a) *P* = 0.009, (b) *P* = 0.000, (c) *P* = 0.000, (d) *P* = 0.013, (e) *P* = 0.167. (f) Categories and associated *P*-values; 1–4 (*P* *=* 0.361), 5 (*P* = 0.049), 6 (*P*  =0.806), 7 (*P* = 0.584), 1–100 µm (*P* = 0.340), 100–200 *µ*m (*P* = 0.246), 200–300 *µ*m (*P* = 0.045), 300 *µ*m to 1 mm (*P* = 0.000), and >1 mm (*P* = 0.000).

In response to 8-d growth on K^+^-deprived media, Col-0 seedlings showed a small reduction in the length of the PR (Figure 1A), but a large significant reduction in the number of emerged LRs (Figure 1B) and in the length of the LRs (Figure 1C;Supplemental Figure S1). Light microscopy was used to characterize LR development before emergence from the PR. Auxin accumulation occurs at the sites of new LRs (Benková et al., 2003), and by using the auxin-responsive *DR5::GUS* reporter, visualization of the early stages of LR development is facilitated (Stages 0–5, defined by Malamy and Benfey, 1997). Stages 1–4 occur before LR primordia (LRP) transverse the endodermis and Stages 5–8 take place after the endodermis has been crossed. Stage 8 marks the emergence from the PR and has been characterized here within the 0–100 *µ*m category. Early-stage LRs can be seen as foci of blue staining along the PR (Figure 1G). There was a reduction in the number of initiating LRs under K^+^-deprivation compared with K^+^-sufficient conditions (Figure 1D), consistent with other observations (Shahzad et al., 2020); however, the reduction in the total number of LRs initiated was less than the reduction in LRs that show emergence (Figure 1B). Density of LRs was calculated by dividing the LR number by the PR length, and no difference between the two K^+^ conditions was observed (Figure 1E), suggesting that the difference in LR number is linked to the reduced PR length. These data show that K^+^-deprivation causes an attenuation of LR growth at an early stage of development, rather than LR initiation, under the conditions used.

The transition from LRP to LR occurs after the formation of the functional LR meristem, at which point the LR grows via cell divisions at the LR root apex as opposed to division of the basal cells as occurs at earlier stages, corresponding to an LR length of *∼*100–200 *µ*m (Malamy and Benfey, 1997). To characterize the stage at which development was inhibited by K^+^-deprivation, the number of LRs and LRP were counted at each stage of development along each PR after 8-d growth on 2or 0.005 mM K^+^. The average number of LRs at each stage of development remained approximately the same between the two conditions until after emergence from the PR (Figure 1F). Following emergence, the number of LRs longer than 1 mm was significantly greater in the K^+^-sufficient treatment than in the K^+^-deprived treatment, with a larger number of the LRs under K^+^-deprivation in the 200 *µ*m to 1 mm categories (Figure 1F). These results suggest that inhibition by K^+^-deprivation takes place after the development of the LR meristem. The elongation of the LRs appears to be adversely affected by K^+^-deprivation as there was a higher number of LRs in categories < 1 mm, whereas under K^+^-sufficient conditions, most LRs elongate ˃1 mm (Figure 1F).

### LR meristem size is reduced under K^+^-deprivation

**Figure 2:**
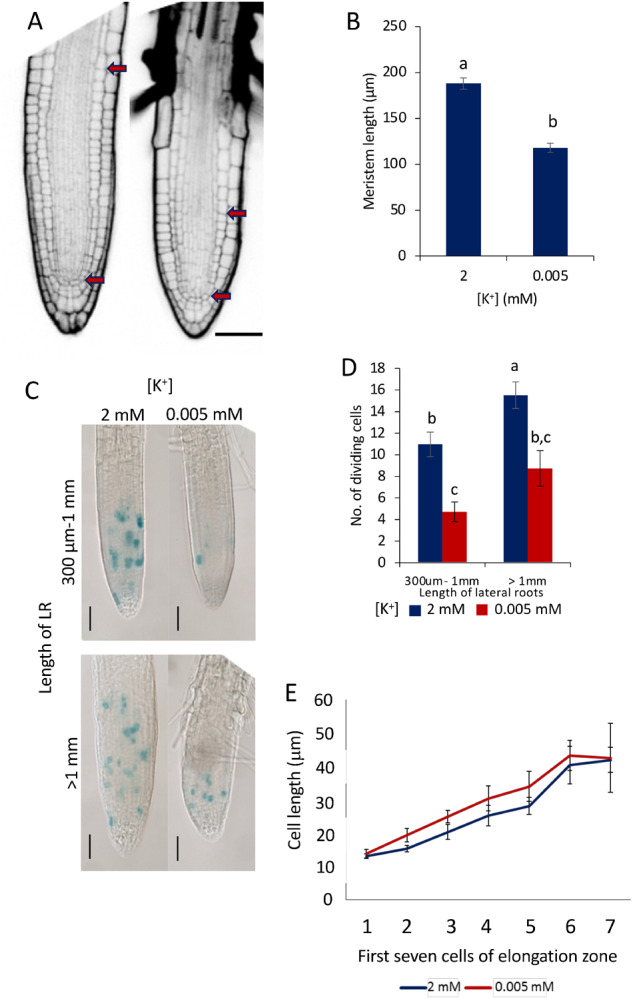
Effect of K^+^-deprivation on LR meristem activity. A, LR meristems of Col-0 seedlings grown for 4 d on half MS10 then 8 d on 2 mM [K^+^] (left) or 0.005 mM (right) [K^+^]. Arrows denote meristem size. Scale bar = 50 *µ*m. B, Length of meristems in LRs of between 300 *µ*m and 1 mm in length. The meristem border is defined as the region of isodiametric cells from the QC up to the cell that was twice the length of the immediately preceding cell (calculated from the cortex cell layer). Values represent means± se, *n* ≥ 18 taken from at least 15 individual seedlings. Letters indicate significance with independent samples *t* test (*P* < 0.05) (*P* = 0.000). C, *CYCB1;2:GUS* expression in seedlings grown for 4 d on half MS10 followed by 8 d K^+^ treatment (2 or 0.005 mM)*;* the GUS staining reveals a reduced area of cell division in 0.005 mM [K^+^]; scale bars = 50 *µ*m. D, Mean number of dividing cells recorded as cells stained blue in the *CYCB1;2:GUS* line. Values are means ± se, *n*  ≥ 32, from at least 10 individual seedlings. Letters indicate significance with a Tukey pairwise comparison *P* < 0.05. E, Lengths of the first seven cells of the elongation zone of LRs grown for 4 d half on MS10 followed by 8 d on 2 or 0.005 mM [K^+^]. Measurements taken from at least six different seedlings, *n*  ≥ 11 for all apart from 7, where *n* = 6. Values are means ± se. Independent sample *t* tests found no significance between [K^+^] for any of the cells (*P* < 0.05). 1 (*P* = 0.597), 2 (*P* = 0.1), 3 (*P* = 0.121), 4 (*P* = 0.306), 5 (*P* = 0.268), 6 (*P* = 0.695), 7 (*P* = 0.953).

Growth of the PR and LRs is maintained by controlling the rates of cell division, elongation, and differentiation. A reduction in LR growth in response to K^+^-deprivation could therefore be regulated by modulation of meristem activity. Confocal imaging was used to investigate meristem size in LRs grown in sufficient- or deprived-K^+^ conditions. The meristem size was calculated as the region of isodiametric cells extending from the quiescent center (QC) to the cell that was twice the length of the immediately preceding cell (González-García et al., 2011). The boundary of the transition zone is different in each cell type, and therefore in all analyses undertaken here, the cortex cell file was used to define the boundary. The length of the meristematic zone in the LRs was reduced after 8 d K^+^-deprivation (Figure 2, A and B).

The frequency of cells entering mitosis in LRs was investigated under sufficient and deprived K^+^ conditions using the *CYCB1;2::GUS* marker, expressed during the G2 to M transition of the cell cycle and an established proxy for determining cell division (Schnittger et al., 2002). GUS-positive cells in the LR meristems were counted as a measure of the number of cells entering mitosis. A reduced number of GUS-positive cells in response to K^+^-deprivation were observed in LRs between 300 *µ*m to 1 mm and also ˃1 mm in length (Figure 2, C and D). This suggests that the reduced meristem size and growth of the LRs is, at least in part, due to a reduced cell division activity.

After undergoing division in the meristematic zone of the root, cells enter the transition/elongation zone where they begin to elongate. The lengths of the first seven cells of the elongation zone were measured to determine the elongation rate in response to K^+^-deprivation. No significant difference was seen in the length of the first seven cells between the sufficient and deprived K^+^ conditions after 8-d growth (Figure 2E), suggesting that the elongation rate is not affected by K^+^-deprivation.

### Identity of the QC is maintained in LRs under K^+^-deprivation

The reduced meristematic cell division activity could be associated with a loss of QC identity or function causing the surrounding stem cells to differentiate, resulting in growth arrest of the LR. To investigate whether the activity of the LR stem cell niche is still maintained under 8 d K^+^-deprivation, the promoter activity of the QC-specific markers *proWOX5::GFP* and *QC25::GUS* were investigated using fluorescence microscopy and histochemical staining, respectively (Sabatini et al., 2003; Sarkar et al., 2007). *WOX5* promoter activity was detected in all LRs ˃200 *µ*m, in all seedlings (*n* = 9), under both sufficient and deprived K^+^ conditions. Confocal microscopy showed no difference in *proWOX5::GFP* expression pattern between the different K^+^ conditions (Supplemental Figure S2B). Histochemical staining of *QC25::GUS* showed no difference between treatments in LRs ˃1 mm (*n* > 10; Supplemental Figure S2C). WOX5 acts to initiate and maintain identity of the QC (Forzani et al., 2014), and so its continued expression, and that of *QC25*, suggests that the identity of the QC is maintained under K^+^-deprivation.

### Gene expression profiling to identify potential regulatory hormonal signaling pathways

**Figure 3:**
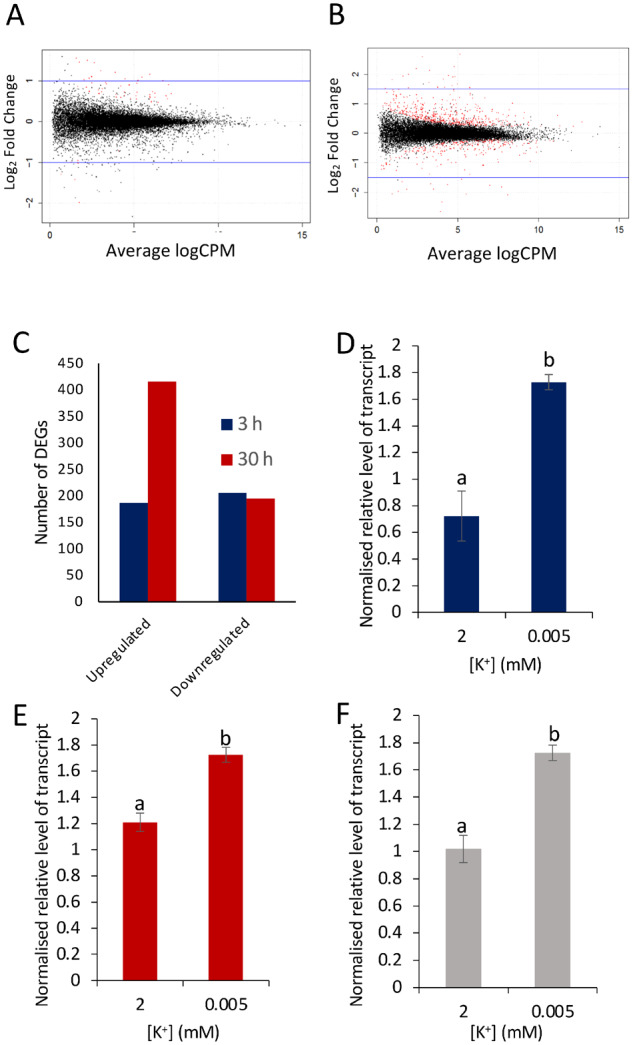
DEGs in seedlings grown on 0.005 mM (K^+^-deprived) or 2 mM (K^+^-sufficient) for 3 or 30 h, identified through RNA-Seq. A, Data represent the outputs from three independent biological replicates per sample; *P* ≤0.05 and a log_2_ fold change (log_2_fc) ≥0.5 and FDR < 0.05. Red dots are significant DEGs, black dots are nonsignificant; counts per million after 3 h (A) or 30 h (B) K^+^-deprivation. C, Histogram of significant DEGs separated into up- and downregulated genes. D–F, Relative normalized levels of transcripts of *HAK5* (D), *ERF6* (E), and *STZ* (F) after 30 h K^+^ treatment (2 mM or 0.005 mM [K^+^]), determined by RT-qPCR. Expression levels were normalized against *AT1G13320*; values are means ± se for three biological repeats with three technical repeats. Letters indicate significance between 2 and 0.005 mM with independent samples *t* test, *P* < 0.05.

Root architectural changes are mediated by the combined actions of different hormones (Bellini et al., 2014; Moore et al., 2015). Therefore to identify hormonal signaling pathways that might play a role in the reduced LR growth response, RNA-Seq was used to identify changes in gene expression following K^+^-deprivation. Samples were taken at 3 and 30 h after transfer of seedlings to either sufficient or deprived K^+^ media (2 or 0.005 mM K^+^), and a *P* ≤0.05, a log_2_ fold change (log_2_fc) >0.5 or ≤−0.5 and an false discovery rate (FDR)  < 0.05 were selected to identify differentially expressed genes (DEGs) between sufficient and deprived K^+^ conditions (Figure 3, A and B; Supplemental Table S1). In total, 416 genes were upregulated and 195 downregulated after 30 h K^+^-deprivation (Figure 3C). These low numbers of DEGs and relatively small fold changes are consistent with previously published microarray data showing that, unlike nitrate or phosphate deficiency, K^+^-deprivation does not lead to major alterations in transcript abundance (Maathuis et al., 2003; Gierth et al., 2005; Ma et al., 2012). To verify the data produced by the RNA-Seq experiment, RT-qPCR was conducted on three DEGs identified from the RNA-Seq data (Supplemental Table S1), namely *HAK5* (upregulated at 3 h, log_2_fc 0.92; *P* = 0.026; upregulated at 30 h, log_2_fc 2.60; *P* = 2.35E-31), *ETHYLENE RESPONSIVE ELEMENT BINDING FACTOR* 6 (*ERF6*; upregulated at 3 h, log_2_fc 0.93; *P* = 1.49E-07; upregulated at 30 h, log_2_fc 0.84; *P* = 0.0001) and *STZ* (upregulated at 3 h, log_2_fc 0.83; *P* = 0.003; upregulated at 30 h, log_2_fc 0.79; *P* = 0.012). RT-qPCR analysis of these genes corresponded with the RNA-Seq data, with all genes showing significant upregulation in response to K^+^-deprivation (Figure 3, D–F).

We carried out gene ontology (GO) analysis of the RNA-seq data (Supplemental Figures S3–S6). The treemaps show a strong upregulation of genes involved in the response to stress and pathogens following transfer to low K^+^, as seen by “response to chitin” being the largest supercluster in each of the upregulated treemaps (Supplemental Figures S3 and S5). Both upregulated DEG treemaps (3 h up and 30 h) also hold superclusters designated “immune system process,” “response to stimulus” and “multi organism process,” and revealed enrichment in the biosynthesis of the hormone ethylene (Supplemental Figures S3 and S5). Auxin biosynthesis showed downregulation at 3 h (Supplemental Figure S4), and a large cluster of genes associated with transcription and photosynthesis and other aspects of metabolism were downregulated at 30 h (Supplemental Figure S6). As hormones are the key in the orchestration of many developmental processes and responses to biotic and abiotic stresses, the overrepresentation of these GO terms suggests that hormonal pathways and ROS are altered in response to K^+^ starvation.

### Auxin plays a necessary but insufficient role in the LR growth response to K^+^-deprivation

Auxin plays an essential role in the control of meristem size and growth of the root, and it promotes cell division and cell elongation and inhibits differentiation (Dello Ioio et al., 2007, 2008; Moubayidin et al., 2010; Perrot-Rechenmann, 2010), whilst at high concentrations it inhibits root elongation (Eliasson et al., 1989). Auxin also controls the development of LRs through multiple auxin-signaling modules (Lavenus et al., 2013). A reduction in both the concentration of free indole-3 acetic acid (IAA) and in basipetal auxin transport has been found in the PRs of seedlings subjected to K^+^-deprived conditions (Shin et al. 2007). Recently, Shahzad et al. (2020) found that genetic variation in the chromatin remodeling factor CLSY1 is associated with reduced LR development in response to low K^+^ via effects on the expression of *IAA27*, encoding a negative regulator of auxin responses and LR development.

GO analysis of the RNA-seq data revealed no clear pattern of transcriptional change in auxin biosynthesis, degradation, or signaling genes at either 3 or 30 h after treatment (Supplemental Figures S3–S6). For example, downregulation of the auxin biosynthesis gene *YUC8* (Hentrich et al., 2013) occurred after 3 h K^+^ starvation (log_2_fc −0.59; *P* = 0.002). Two cytochrome P450-encoding genes (*CYP79B3* and *CYP79B2*) involved in the conversion of tryptophan to indole-3 acetaldoxime (IAOx; Zhao et al., 2002) were downregulated after 3 h (*CYP79B3*: log_2_fc −0.69; *P* = 0.049; *CYP79B2*: log_2_fc −0.76; *P* = 0.026). The *IAA-METHYLTRANSFERASE-1* gene, which plays a role in auxin homeostasis in converting IAA into the nonpolar inactive form methyl-IAA (Qin et al., 2005; Li et al., 2008), was also downregulated (at 3 h, log_2_fc−0.55; *P* = 0.004). However, the most highly upregulated gene after the 30 h treatment (*CYP71A12*; log_2_fc 2.68; *P* = 8.43E-30) was identified as acting in the conversion of IAOx to indole-3-acetonitrile (IAN). There was also downregulation of a number of auxin-responsive genes after 3 h (*SAUR20* [log_2_fc −1.02; *P* = 7.55E-05]*, SAUR24* [log_2_fc−0.77; *P* = 0.009]*, SAUR22* [log_2_fc−0.82; *P* = 0.008], and *IAA29* [log_2_fc−0.59; *P* = 0.016]); however, this downregulation was not seen after 30 h.

Some genes associated with auxin signaling were upregulated in response to K^+^ starvation. These included *PINOID-BINDING PROTEIN 1*, a calcium-binding protein that interacts with PINOID (a key component of auxin signaling) and upregulated by auxin (Benjamins et al., 2003), which was upregulated after 3 h (log_2_fc 0.87, *P* = 6.6E-05) and also, but to a lesser extent (log_2_fc 0.64, *P* = 0.002), after 30 h. The genes encoding the Broad-Complex, Tramtrack and Bric a brac (BTB) and Transcription Adaptor putative Zinc finger (TAZ) domain proteins 2 and 5 (*BT2* and *BT5*) were, respectively, upregulated after 3 h (*BT2*: log_2_fc 0.91, *P* = 9.71E-10) and 30 h (*BT5*: log_2_fc 1.01, *P* = 2.26E-16) but have also been linked to other hormonal and abiotic signals as well as auxin (Hammer et al., 2009; Mandadi et al., 2009; Canales et al., 2014), making it difficult to link them directly to auxin.

**Figure 4:**
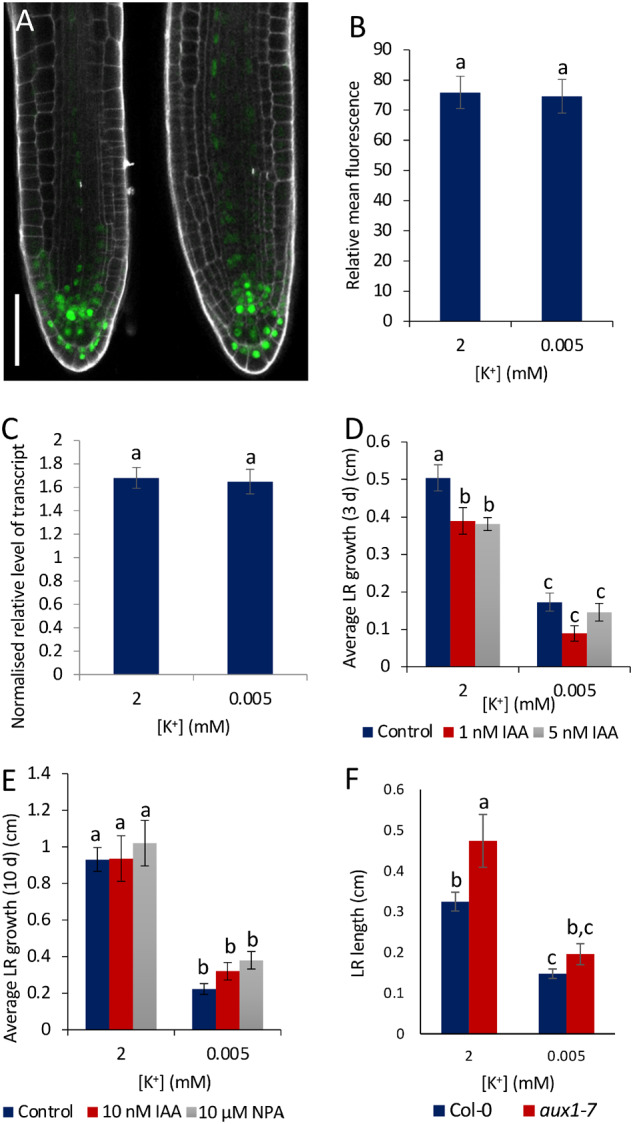
The role of auxin in the reduced LR growth response to K^+^-deprivation. A, Representative fluorescence in LRs ˂1 mm in length of *pDR5rev::3xVENUS-N7* seedlings after 4 d growth on half MS10 followed by 8 d grown on 2 mM (left) or 0.005 mM (right) [K^+^]. Scale bar = 50 *μ*m. White is PI stain, green is VENUS. B, Relative mean fluorescence of LRs of each treatment measured using ImageJ. Images taken from at least 8 different seedlings per treatment, *n*  ≥ 14 LRs. Values are means ± se. Letters indicate significance as calculated with an independent sample *t* test, *P* = 0.159. C, Normalized relative level of *IAA2* transcripts after 72 h K^+^ treatment (2 or 0.005 mM) determined by RT-qPCR. Samples taken from seedlings 14 DAG, normalized against *AT1G13320.* Values are means ± se. Three biological repeats and three technical repeats were used. Independent samples *t* test determined there was no significant difference between treatments, as denoted by letters (*P* <0.05). D, Mean LR growth over 3 d following [K^+^] treatment (2 or 0.005 mM in the presence of 0, 1, or 5 nM IAA), 12 DAG. E, Mean LR length after 10 d treatment with 0 and 10 nM IAA or 10 μM NPA, 19 DAG. Values are means of at least eight individual seedlings per sample, ±se for (D, E). Letters indicate significance with a Tukey pairwise comparison, *P* < 0.05. F, Mean LR growth after 8 d K^+^ treatment (2 or 0.005 mM), 12 DAG seedlings of Col-0 and *aux1-7.* Values are means of at least 25 individual seedlings ±SE, *n* ≥ 25. Letters indicate significance with a Tukey pairwise comparison, *P* < 0.05.

A potential role for auxin in the reduced LR growth response was investigated further. Expression of the auxin reporter *DR5rev::3XVENUS-N7* (Heisler et al., 2005) in LRs of seedlings grown on sufficient or deprived K^+^ conditions was used as a proxy for monitoring potential changes in auxin distribution. No detectable differences were observed in either the pattern or level of expression of the reporter in LRs of ˂1 mm when seedlings were grown under the different K^+^ conditions (Figure 4, A and B). Analysis of the expression of the auxin-responsive gene *IAA2* by RT-qPCR in seedlings grown for 72 h on sufficient or deprived K^+^ conditions similarly showed no significant difference (Figure 4C).

To determine whether exogenous auxin treatment could rescue the short LR phenotype of K^+^-deprived seedlings, the growth medium was supplemented with 1 or 5 nM IAA for a period of 3 d (Figure 4D), or 10 nM IAA or 10 *μ*M NPA for a period of 10 d (Figure 4E). Addition of IAA did not alter LR development under K^+-^-deprived conditions when compared with the nonsupplemented media (Figure 4, D and E). LR growth was also not restored in either the presence of NPA, an inhibitor of polar auxin transport (Figure 4E) or in the auxin transport-defective *aux1-7* mutant (Figure 4F), suggesting that enhanced auxin transport does not play a determinative role in the reduced LR growth response to K^+^-deprivation. Furthermore, analysis of expression of the *CYCB1;2:GUS* reporter showed that addition of 1 or 200 nM IAA made no difference to the length of the LR meristems when compared with controls (Supplemental Figure S7). These results suggest that the reduction in cell division and meristem size in response to K^+^-deprivation is not associated with reduced auxin content in the meristem.

### Ethylene does not have an essential role in the LR growth response to K^+^-deprivation

The hormone ethylene has been identified as a key hormone in the K^+^-deprivation pathway, with evidence to suggest upregulation in response to K^+^-deprivation (Jung et al., 2009) and downstream regulation of K^+^ transporters and ROS (Jung et al., 2009; Nam et al., 2012). Ethylene is known to inhibit LR growth (e.g. Lewis et al. 2011), but its role in the reduced LR phenotype in response to K^+^-deprivation has not been investigated.

**Figure 5:**
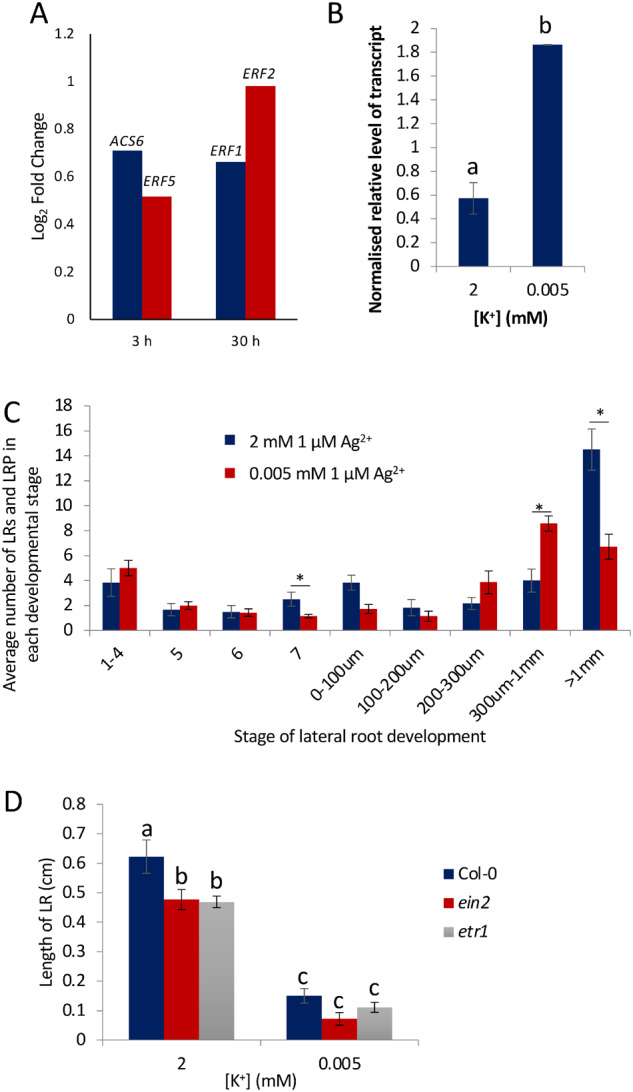
The role of ethylene in the reduced LR growth response to K^+^-deprivation. A, Changes in expression of *ACS6* and *ERF5* after K^+^ treatments at 3 h and *ERF1* and *ERF2* after K^+^ treatments at 30 h. B, Normalized relative level of transcript of *ERF1* after 72 h K^+^ treatment (2 or 0.005 mM [K^+^]). Samples were taken from seedlings 14 DAG, analyzed by RT-qPCR, and normalized against *AT1G13320.* Values are means ± se of three biological repeats with three technical repeats. Letters indicate significance with independent samples *t* test, *P* < 0.05. C, LR progression analysis. Light microscopy and the auxin-responsive *DR5::GUS* reporter line allowed all LRP and LRs to be counted along the length of the PR. Col-0 seedlings were analyzed 12 DAG and after 8 d of K^+^ treatment (2 or 0.005 mM). Primordial stage from 1 to 7 as defined in Malamy and Benfey (1997). Stages 1–4 occur before LR founder cells transverse the endodermis and Stages 5–8 take place after the endodermis has been crossed. Stage 8 marks the emergence from the PR and therefore has been characterized here within the 0–100 *µ*m category. Media were supplemented with 1 *μ*M Ag^2+^. Values are averages taken from at least seven individual seedlings ±se. Asterisks indicate significance with independent samples *t* test, *P* < 0.05). D, Mean length of LRs after 8 d K^+^ treatment (2 or 0.005 mM) 12 DAG of seedlings of Col-0 and ethylene mutants *ein2* and *etr1-1* values are means of at least 10 individual seedlings ±SE. Letters indicate significance with a Tukey pairwise comparison, *P* < 0.05.

GO analysis of the RNA-seq data showed enrichment, in the upregulated data, of a number of ethylene-related terms from the RNA-Seq data, including ethylene biosynthesis (3 and 30  h), ethylene-activated signaling pathway (3 h), response to ethylene (3 and 30 h) and cellular response to ethylene (30 h; Supplemental Figures S3 and S4). Therefore, the transcriptional regulation of the ethylene biosynthesis pathway was investigated. A gene encoding a key enzyme in the ethylene biosynthetic pathway (*1-AMINOCYCLOPROPANE-1-CARBOXYLIC ACID SYNTHASE 6* [*ACS6*]) was upregulated by log_2_fc 0.71 (*P* = 0.002) after 3 h low K^+^ treatment (Figure 5A), but was not significantly different from the control conditions after 30 h, suggesting that the upregulation of ethylene is a rapid and transient reaction to low K^+^ treatment.

A number of *ETHYLENE RESPONSIVE ELEMENT BINDING FACTOR* (*ERF)* genes induced by ethylene treatment were also upregulated in response to K^+^ starvation; *ERF5* at 3 h (log_2_fc 0.52; *P* = 0.016), *ERF1* (log_2_fc 0.66; *P* = 8.53E-07), and *ERF2* (log_2_fc 0.98; *P* = 3.75E-07) at 30 h (Figure 5A). Analysis of the expression of *ERF1* by RT-qPCR in seedlings grown for 72 h on either low or standard K^+^ conditions also showed significantly higher expression of *ERF1* under low K^+^ conditions (Figure 5B). Other upregulated genes identified by the GO analysis as relating to ethylene signaling were several WRKY and NAM, ATAF1/2 and CUC2 (NAC) TF genes (Supplemental Figure S2 and Supplemental Table S2).

To investigate whether increased ethylene signaling and biosynthesis might affect LR growth in response to K^+^-deprivation, LRs and LRP numbers at each stage of development were determined under sufficient and deprived K^+^ conditions when ethylene responses were inhibited by the presence of 1 *μ*M Ag^2+^ (Figure 5C). Analysis was carried out after 8-d growth on sufficient and deprived K^+^ treatment, and ethylene inhibition had no significant effect on the root growth pattern when compared to wild-type (WT), that is, displaying a larger number of LRs elongating past 1 mm in the sufficient compared with the deprived K^+^ conditions (Figure 5C). LR length was measured in the mutants *ethylene insensitive2* (*ein2*) and *ethylene resistant1-1* (*etr1-1*) after 8 d K^+^-deprivation, and no rescue of LR growth was observed in the length of K^+^-deprived LRs in the mutants compared with WT (Figure 5D). This suggests that ethylene does not play a major role in reducing LR growth in response to K^+^-deprivation.

### The GA pathway is implicated in the response to K^+^-deprivation

The gibberellic acid (GA) pathway was identified from the RNA-Seq data as potentially regulated during the early stages of the K^+^-deprivation response. GA homeostasis is controlled through its biosynthesis and deactivation, with three families of dioxygenases, namely the *GIBBERELLIN-3-OXIDASES* (GA3oxs), GA20oxs, and the GA2oxs playing a key role in the regulation of this pathway in response to many developmental and environmental cues (Colebrook et al., 2014). The GA biosynthesis gene *GA3ox2* was downregulated after 30 h K^+^-deprivation (log_2_fc −0.51; *P* = 0.001), and two TFs with known roles in repressing the GA signaling pathway were identified from the upregulated data [JUNGBRUNNEN1 {*JUB1*} and *ERF6*] (Supplemental Figure S8A). *ERF6*, which was transcriptionally upregulated (log2fc 0.84; *P* = 0.001) in response to K^+^-deprivation (Supplemental Figure S8A), has been suggested to activate *GIBBERELLIN-2-OXIDASE 6* (*GA2ox6*), a GA deactivation gene (Dubois et al., 2015); and *JUB1* (upregulated by log2fc 1.33; *P* = 2.78E-10; Supplemental Figure S8A) interacts with a number of GA -related genes (Supplemental Figure S8B). For example, JUB1 represses *GIBBERELLIN 3 BETA-HYDROXYLASE 1* (*GA3ox1*; Supplemental Figure S8B), a gene involved in the later steps of the GA biosynthetic pathway, and also activates the negative regulators of GA responses *REPRESSOR OF GIBBERELLIC ACID1-LIKE1* (*RGL1*) and *GIBBERELLIN INSENSITIVE* (*GAI*) (Supplemental Figure S8B; Shahnejat-Bushehri et al., 2016, 2017), both encoding members of the DELLA family of proteins that restrain cell proliferation and expansion (Achard and Genschik, 2009). One other negative regulator of GA responses, GASA5 (Zhang et al., 2009), was the second most upregulated gene at 3 h in the RNA-seq data set (log_2_fc 1.56; *P* = 2.46E-05), but was not significantly differentially expressed at 30 h (Supplemental Table S1).

**Figure 6:**
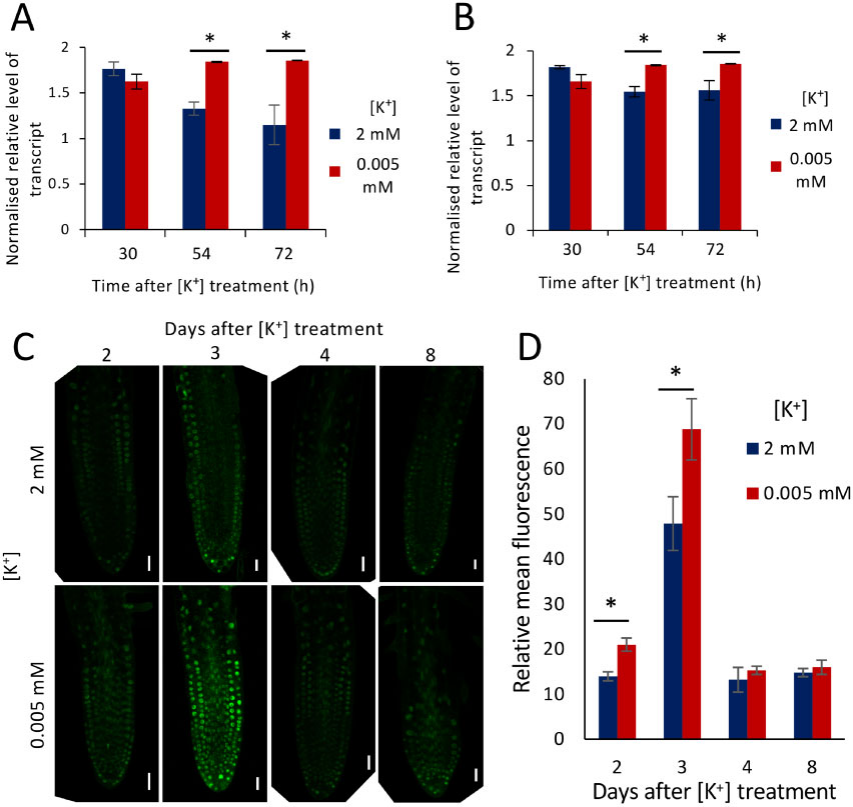
The role of GA in the reduced LR growth response to K^+^-deprivation. A, B, Effect of K^+^-deprivation on GA-related gene expression. Transcription of *GA2ox6* (A), *GA3ox1* (B) after 30, 54, and 72 h K^+^ treatment (2 or 0.005 mM), determined by RT-qPCR and normalized against *AT1G13320.* Seedlings were grown for 11 d on half MS10 followed by transfer to K^+^ treatment. Values are means ± SE of three biological repeats with three technical repeats. Asterisks indicate significance with independent samples *t* test, *P* < 0.05. C, *proRGA::GFP:RGA* protein localization in LRs grown for 9 d on half MS10 followed by 2 mM (top panels) or 0.005 mM (bottom panels) [K^+^] for 2, 3, 4, or 8 d; scale bars = 20 *μ*m. D, Relative mean fluorescence of RGA GFP in LRs after 2, 3, 4, or 8 d K^+^ treatment (2 or 0.005 mM). LRs ˂1 mm were analyzed for all treatments apart from 8 d where LRs under and ˃1 mm were analyzed. Images taken from at least six different seedlings; values represent means ± SE. Asterisks indicate significance from independent samples *t* test, *P* < 0.05.

To investigate further if K^+^-deprivation causes transcriptional changes that could affect GA responses, the expression levels of the GA biosynthesis gene *GA3ox1*, and the GA deactivation gene *GA2ox6* were quantified using RT-qPCR after 30, 54, and 72 h of K^+^-deprivation (samples taken from seedlings at 12, 13, and 14 DAG, respectively). The expression of the GA deactivation gene, *GA2ox6*, was upregulated after 54 and 72 h of K^+^-deprivation (Figure 6A), consistent with an increase in the deactivation of bioactive GAs in response to K^+^-deprivation. Similarly, the *GA3ox1* biosynthesis gene was upregulated after 54 and 72 h K^+^-deprivation (Figure 6B). These data suggest that after 30 h, there is transcriptional response to K^+^-deprivation that may lead to reduced GA signaling.

A decrease in GA signaling leads to the stabilization of growth inhibitory DELLA proteins (Sun and Gubler, 2004), and so we investigated expression of the DELLA gene fusion *proRGA::GFP:RGA* (Silverstone et al., 2001) in LRs in response to K^+^-deprivation. Seedlings were grown for 9 d on half-strength Murashige and Skoog ( half MS10) growth medium containing sufficient K^+^, followed by 2, 3, 4, and 8 d on either K^+^-deprived or K^+^-sufficient media before imaging. Average relative fluorescence levels of *proRGA::GFP:RGA* were significantly higher in LRs of seedlings that had been exposed to K^+^-deprived conditions for 2 and 3 d compared to the seedlings grown on K^+^-sufficient medium (Figure 6, C and D), showing that there is a stabilization of DELLA proteins in LRs in response to K^+^-deprivation. Interestingly the increase in DELLA levels was not seen after 4 or 8 d K^+^-deprivation (Figure 6, C and D), suggesting that the DELLA stabilization is transient. Although most DELLA genes are not upregulated at the transcriptional level in response to stress (Colebrook et al., 2014), we used RT-qPCR to determine whether any upregulation of the genes encoding GAI, RGA, RGL1, RGL2, and RGL3 could be detected (Supplemental Figure S9). Although there was a trend in upregulation of RGA, RGL1, and RGL2 at 30 h on low K^+^ medium, the differences were not statistically significant, in agreement with the RNA-seq data.

### Exogenous GA restores the length of LR meristems and growth under K^+^-deprivation

**Figure 7:**
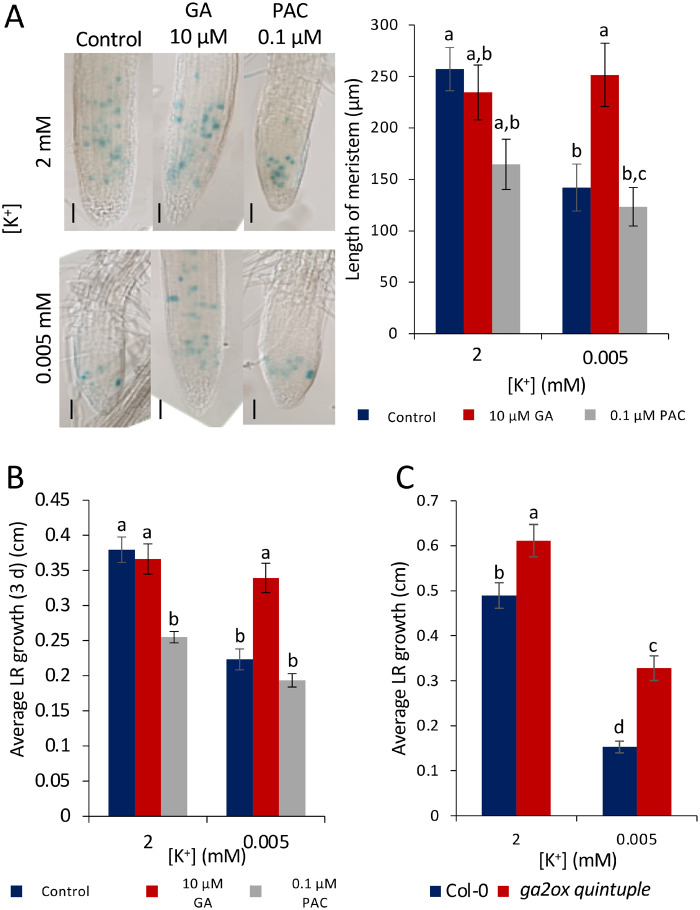
Effect of GA on meristem activity under K^+^-deprivation. A, *CYCB1;2:GUS* expression (GUS histochemistry) shows a reduced area of cell division in reduced [K^+^]; scale bars = 50 µM; length of meristem measured as the length of root with dividing cells. LRs > 1 mm. Media were supplemented with either 10 *µ*M GA or 0.1 *µ*M PAC for 8 d. Analysis was carried out on seedlings 12 DAG. Values are mean ± SE taken from at least six individual seedlings per treatment. B, Mean LR growth over 3 d on 2 or 0.005 mM K^+^ treatment. Seedlings were grown for 9 d on half MS10 before movement to K^+^ treatment. Values are means ± SE taken from at least 17 individual seedlings per treatment. C, Mean LR growth of Col-0 or the *ga2ox quintuple* mutant (Rieu et al., 2008) grown on 2 or 0.005 mM K^+^ for 3 d. Seedlings were analyzed 12 DAG. Values are means ± se of at least 10 individual seedlings per treatment. For all panels, letters indicate significance with a Tukey pairwise comparison, *P* < 0.05.

DELLA proteins are involved in reduction of growth by restraining cell proliferation and expansion (Peng et al., 1997, 1999; Fleet and Sun, 2005). We therefore hypothesized that the reduction in LR growth in response to K^+^-deprivation could be due to a reduction in GA signaling leading to a stabilization of DELLAs. This hypothesis was investigated by measuring meristem size, using *CYCB1;2::GUS* expression as a cell division marker (Figure 7A), with and without exogenous GA application under K^+^-deprivation. Seedlings were grown for 4 d on half MS10 followed by 8 d on K^+^-deprived or K^+^-sufficient medium, in the presence of either GA or the GA synthesis inhibitor paclobutrazol (PAC). In the LRs of length >1 mm, the application of 10 *µ*M GA restored the size of the LR meristem under K^+^-deprivation, and application of 0.1 *µ*M PAC led to a reduced LR length under the K^+^-sufficient conditions (Figure 7A). Similar trends were seen in shorter LRs (100 *µ*m to 1 mm), but the differences were not statistically significant (Supplemental Figure S10).

Average LR growth was also measured in roots grown in the presence or absence of either 10 *µ*M GA or 0.1 *µ*M PAC. Seedlings were grown on half MS10 for 9 d, then transferred to K^+^-deprived or K^+^-sufficient media for 3 d. These results mirrored the observations for the effects on meristem size (Figure 7B), with LR growth restored with the addition of GA, and LR growth restricted in the K^+^-sufficient conditions when PAC was added (Figure 7B). Blocking GA deactivation in the *ga2ox* quintuple mutant (Rieu et al., 2008) also partially restored LR growth over 3 d of K^+^-deprivation (Figure 7C).

### LR growth reduction in response to K^+^-deprivation is enhanced in *JUB1* and *CBF1* overexpressers and attenuated in the *sfr6-1* mutant

**Figure 8:**
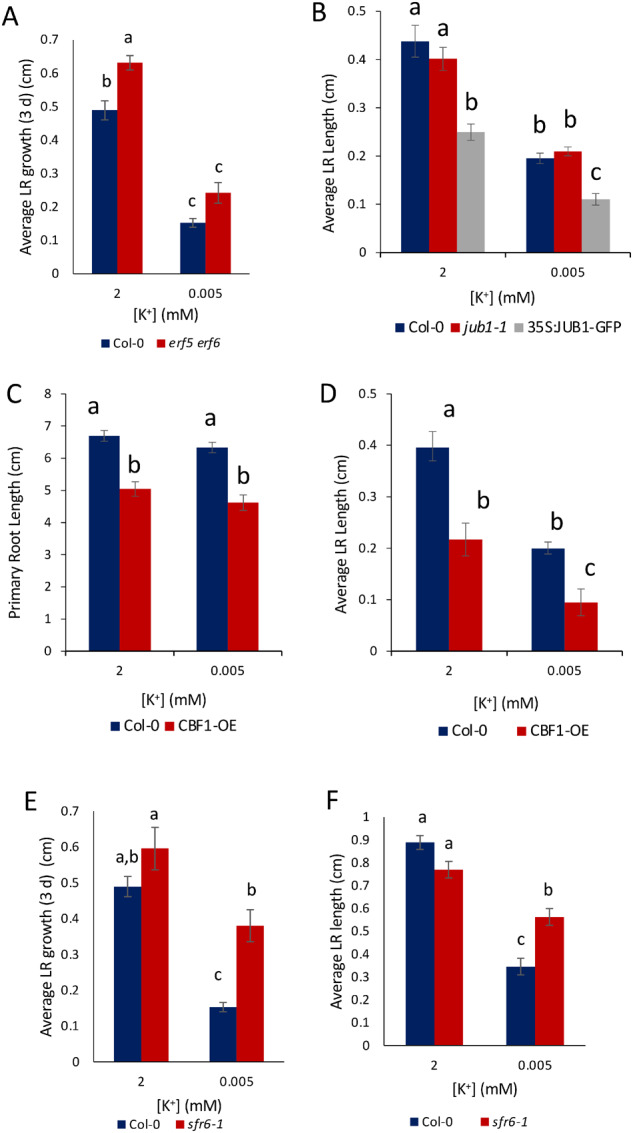
Role of TFs in regulating LR growth under K^+^-deprivation. A, Average LR growth over 3 d K^+^-deprivation in WT and *erf5 erf6* double mutant seedlings. B, Average LR length of WT, *jub1-1 and JUB1* overexpressing seedlings after 8 d K^+^-deprivation. C, D, Mean length of PRs (C) and LRs (D) after 4 d of growth on half MS10 and then 8 d of K^+^ treatment (2 or 0.005 mM) of Col-0 and *CBF1*-overexpressing seedlings. E, F, Average LR growth over 3 d (E) and 8 d (F) K^+^-deprivation in WT and *sfr6-1* mutant seedlings. Values are averages taken from nine individual seedlings (A, B), 17 seedlings (C, D), and 21 seedlings (E, F) ±se. Letters indicate significance with a Tukey pairwise comparison, *P* < 0.05.

To elucidate potential upstream regulators of the GA response, the roles of a number of TFs in the reduced LR growth response were investigated. JUB1 (NAC042), ERF6, and C-repeat-binding factor (CBF1) were studied further because of their transcriptional upregulation in response to K^+^-deprivation (Supplemental Figure S8A) and known roles in repressing GA signaling pathways (Wu et al., 2012; Dubois et al., 2013; Shahnejat-Bushehri et al., 2016, 2017; Thirumalaikumar et al., 2018). It was hypothesized that the transcriptional increase in one of these TFs could lead to stabilized DELLAs and therefore reduced LR growth. The role of ethylene-related ERF6 was investigated by conducting LR growth analysis of the double mutant *erf5 erf6* because of known redundancies between the *ERF5* and *ERF6* genes (Moffat et al., 2012). Under 3 d, K^+^-deprivation LR growth was not restored in the *erf5 erf6* mutant (Figure 8A), suggesting that these genes do not play a role in the reduced LR growth phenotype. The 8-d analysis was disregarded due to severe stunting of seedlings, suggesting alternative stress pathways were being stimulated. These results also support previous work suggesting that ethylene does not play an important role in the reduced LR growth response to low K^+^ (Figure 5).

The NAC TF JUB1 (Wu et al., 2012) was also hypothesized to regulate the response to K^+^-deprivation due to its strong upregulation during K^+^-deprivation (1.33 log2fc at 30 h, *P*-value 2.78E-10; Supplemental Table S1). Analysis of the *jub1-1* mutant phenotype found that LR growth was not restored under 8 d K^+^-deprivation, suggesting loss of function of JUB1 does not influence response to K^+^ (Figure 8B). However, *JUB1* transgenic overexpressors showed a significant reduction in LR length in response to low K (Figure 8B), implicating a role for JUB1 in restricting LR growth under K^+^ stress.

The CBF/dehydration-responsive element-binding factor family is a small family of TFs known to regulate cold stress acclimation through the activation of CBF regulon targets, the *COLD ON-REGULATED* (*COR*) genes, and are known to regulate GA signaling and biosynthesis through regulation of GA dioxygenases and DELLAs (Achard et al., 2008a; Zhou et al., 2017). RNA-Seq data suggest a potential role for the CBF regulon in K^+^-deprivation through the upregulation of *CBF1* gene expression after 3 h K^+^-deprivation (0.80 log_2_fc) and upregulation of two CBF target genes, *COR15A* and *COR15B*, after 30 h K^+^-deprivation (0.70, 0.71 log_2_fc, respectively; Supplemental Table S1). *COR15A* and *COR15B* are activated by the simultaneous expression of both *CBF1* and *CBF3* under cold (Seki et al., 2002; Maruyama et al., 2004; Novillo et al. 2007); therefore, upregulation of both may suggest an overall upregulation of the CBF regulon.

As *CBF1* was upregulated in response to K^+^-deprivation, and its target *GA2ox6* was also upregulated (Figure 6A), the role of this gene in the reduced LR growth phenotype was investigated further. Since *CBF1* is functionally redundant with related family members (Jia et al., 2016), we tested the effect of overexpressing *CBF1* in transgenic plants. While PR length of the overexpresser was not significantly affected by low K^+^ supply, the length of LRs was significantly reduced compared to WT (Figure 8, C and D), consistent with a role for CBF1 in suppressing LR growth under K^+^ stress.

The SENSITIVE TO FREEZING6 (SFR6) protein, otherwise known as MEDIATOR 16, acts downstream of *CBF1* translation to recruit the core Mediator complex to cold-regulated genes in the activation of cold-responsive genes (Knight et al., 2009). We hypothesized that in the *sfr6-1* mutant (Knight et al., 1999), the activation of *GA2oxs* and DELLAs by CBF1 may be blocked in response to K^+^-deprivation. In support of this hypothesis, the reduction in LR growth in response to K^+^-deprivation was attenuated in the *sfr6-1* mutant when LR growth was measured ˃3 and 8 d (Figure 8, E and F). This attenuation of the reduced LR growth response in the *sfr6-1* mutant demonstrates a role for *CBF1* and *SFR6* in the regulation of LR growth in response to K^+^-deprivation.

## Discussion

We show that, in response to K^+^-deprivation, Arabidopsis Col-0 shows a small reduction in the growth of the PR, while the growth of LRs is more reduced (Figure 1). Specifically, LR initiation, LR primordium development, LR emergence, and LR meristem establishment are unaffected by K^+^-deprivation, but the extension growth of the LRs due to meristem activity is impaired (Figure 1). Image analysis and reporter gene studies show that the LR meristem organization remains intact under K^+^-deprivation, while cell division is reduced, leading to a reduction in meristem size, associated with the observed reduced growth (Figure 2).

Previous research has suggested a role for auxin and ethylene in the response to K^+^-deprivation. While these pathways are strongly implicated in the interdependent regulation of LR development in numerous studies (e.g. Lewis et al., 2011; Lavenus et al., 2013), we found no rescue on the short LR phenotype by exogenous auxin (Figure 4; Supplemental Figure S7), and inhibiting ethylene signaling through gene mutation, or supplementing the medium with silver ions, was not able to rescue LR growth under K^+^-deprivation (Figure 5). This suggests that, under the conditions used for these experiments, the reduced LR growth in response to K^+^-deprivation is coordinated through ethylene- and auxin-independent mechanism, or regulation of growth by these pathways is not limiting but over-ridden by limitations in other pathways, such as the GA pathway. Differences in response seen here compared to other studies may be due to factors such as differences in medium composition, with the auxin response being contingent, for example, on Fe availability (Shahzad et al., 2020) or Arabidopsis ecotype (Kellermeier et al., 2013). It is also possible that a transient GA downregulation in specific cell types is sufficient to lead to reduced PIN-FORMED (PIN) protein function, leading to a reduced auxin maximum in the growing LR (Willige et al., 2011). We have shown recently, for example, that the epidermis signals to cells containing PIN proteins in a noncell-autonomous fashion (Short et al., 2018). This illustrates the complexity of response of plants due to environmental and genetic interactions that require further study of spatiotemporal signaling mechanisms in the future.

Gene expression analysis using RNA-Seq and RT-qPCR identified an increase in the expression of the GA deactivation dioxygenase gene, *GA2ox6* (Figure 6A), as well as an early downregulation of the GA biosynthesis gene *GA3ox2* (Supplemental Figure S8A), suggesting that there is a reduction in GA signaling in response to K^+^-deprivation. Increased accumulation of the DELLA fusion protein RGA:GFP in the LRs of seedlings grown under K^+^-deprived conditions (Figure 6, C and D) is proposed to mediate the reduced cell division and LR growth in response to K^+^-deprivation. Both restoration by exogenous GA of LR cell division and growth under K^+^-deprivation, and the effects of the GA inhibitor PAC in phenocopying K^+^-deprivation, supports the hypothesized link between K^+^-deprivation, reduced GA signaling, and reduced LR growth (Supplemental Figure S11).

The data presented here suggest that the reduction in GA responses may be regulated at a number of levels. The first is through increased GA deactivation, as shown through increased expression of *GA2ox6* after 54 and 72 h K^+^-deprivation (Figure 6A). The second level is through reduced biosynthesis of GA; however, the data presented here are not conclusive on that specific point, and this requires further metabolomic work. An early reduction in biosynthesis is suggested by the RNA-Seq data, with a downregulation of the *GA3ox2* biosynthetic gene after 30 h. However, data on the expression levels of *GA3ox1* showed a small increase in expression levels after 54 and 72 h K^+^-deprivation (Figure 6B). Increased DELLA activity increases expression of *GA20ox1* and *GA3ox1* and decreases levels of *GA2ox* through a feedback mechanism (Hedden and Kamiya, 1997; Cowling et al., 1998; Thomas et al., 1999; Xu et al., 1999). *GA3ox1* is a known direct target of DELLA proteins (Zentella et al., 2007), and so this could explain why *GA3ox1* expression increases in response to K^+^-deprivation. This suggests that GA levels could also be controlled through a feedback loop with DELLA proteins in response to K^+^-deprivation.

Data presented here suggest a potential mechanism for GA regulation through JUB1 and the CBF regulon. JUB1 restricts root growth in response to drought by activating growth-inhibitory DELLAs (Thirumalaikumar et al., 2018) and may play a similar role in the response to K^+^ stress. CBF regulon genes regulate GA and DELLAs in response to cold (Achard et al., 2008a; Zhou et al., 2017)—CBF3 through the upregulation of *GA2ox7* and *RGL* (Zhou et al., 2017) and CBF1 through increasing the expression of *GA2ox3, GA2ox6*, and also through the increase in transcript levels of the DELLA gene *RGL3* (Achard et al., 2008a). This provides a potential mechanism linking K^+^-deprivation, TF expression, and GA signaling to LR growth and development.

A reduction in GA levels leading to DELLA accumulation and inhibition of growth has been identified as a key response to salt, cold, osmotic stress, and low phosphate signaling (Achard et al., 2006, 2008a, 2008b; Jiang et al., 2007; Magome et al., 2008; Dubois et al., 2013; Rowe et al., 2016). Each pathway involves GA deactivation as a key point of control, with increased transcriptional regulation of *GA2ox* genes in each pathway (Jiang et al., 2007; Achard et al., 2008a; Magome et al., 2008; Dubois et al., 2013). Work presented in the current paper has identified K^+^-deprivation as another abiotic stress response mediated by increased DELLA accumulation, leading to a reduction in growth. However, this work presents a model where growth is restricted specifically in the meristems of LRs in response to K^+^-deprivation. We were unable to detect significant levels of DELLA gene upregulation under K^+^-deprivation, consistent with previous data that showed most DELLA genes are not upregulated at the transcriptional level in response to stress (Colebrook et al., 2014); though perhaps a very transient change in DELLA gene expression in restricted celltypes would not be readily detectable, or that DELLA protein stabilization occurs, and this requires further analysis. We suggest that the observed transient stabilization of DELLAs is sufficient to arrest cell division for growth, and given the known interaction between auxin and GA signaling (Fu and Harberd 2003), possibly by limiting the auxin transport to, or responsiveness of LR meristems (Willige et al., 2011). This could account for the complex relationship between K availability, GA, ethylene and auxin signaling, and the link with LR development.

The ecological significance of the Col-0 root architectural change in response to K^+^-deprivation has not been investigated, although it has been suggested that LRs are more important than the PR for the uptake of immobile nutrients, such as phosphorus and manganese, from the soil (Liu et al., 2013), while a deeper root system is more important for taking up mobile nutrients such as K^+^ and nitrogen (Maeght et al., 2013). As K^+^ is such an essential nutrient for growth and functioning of a plant, this might explain the trade-off between PR and LR growth, utilizing resources to grow in a way more likely to find the mobile nutrient K^+^, deeper in the soil. We suggest that such a mechanism is mediated by the tissue-specific regulation of DELLA protein accumulation via key stress-related TFs to limit cell division activity in LRs in response to K^+^-deprivation.

## Methods

### Plant material

Arabidopsis (*A. thaliana*) WT seeds Col-0 were from laboratory stocks originally from Lehle Seeds (Round Rock, TX, USA). All mutants and reporters were in the Col-0 background, obtained from lab stocks unless otherwise stated. *proRGA::RGA::GFP* seed (Silverstone et al., 2001) was courtesy of Ari Sadanandom (Durham University, UK); *QC25::GUS* courtesy of Ben Scheres (Wageningen University, the Netherlands); *WOX5::GFP* courtesy of Chunli Chen (Huazhong Agricultural University, Wuhan, China); *CYCB1;2:GUS* was obtained from the Nottingham Arabidopsis Stock Center; *ga2ox* quintuple mutant was courtesy of Steve Thomas (Rothamsted Research, Harpenden, UK; Rieu et al., 2008); *jub1-1* and the JUB1 overexpresser courtesy of Salma Balazadeh (University of Potsdam, Germany; Wu et al., 2012); *sfr6-1*, *erf5 erf6* and *CBF1* overexpresser courtesy of Marc Knight and Heather Knight (Durham University, UK; Knight et al., 2009).

### Plant growth conditions

Seeds were surface-sterilized for 1 min with 70% (v/v) ethanol, 15 min with 20% v/v commercial bleach with a drop of 0.1% v/v Tween-20, then rinsed 4 times with sterile deionized water. Seeds were stratified for 4–7 d in the dark at 4°C to encourage and synchronize germination. Seedlings were grown for 4 d on horizontal sterile 10 × 10 cm square Petri dishes containing 50 mL of solid half MS10 growth medium (see below for details) containing an adequate supply of K^+^ so as not to stunt initial development and to allow selection of seedlings with synchronized germination times. Plates were sealed around the edges with Micropore™ tape. Seedlings were then transferred to vertical 10 × 10 cm square Petri dishes containing 50 mL of solid K^+^ media for a further 8 d, or vertical half MS10 plates for a further 5 d before being moved to K^+^ media for time-course experiments. For RNA-Seq and RT-qPCR, seedlings were grown for 4 d on horizontal plates then for 7 d on half MS10 vertical plates before transfer to K^+^ media. Tissue was collected for RNA extraction after 3 and 30 h. The vertical 10 × 10 cm Petri dishes were placed vertically in cardboard racks constructed to allow light to the shoots but not to the roots. All experiments were conducted in growth rooms or growth cabinets under long-day conditions (16 h light: 8 h dark) at 22°C.

### Culture media

Half-strength Murashige and Skoog medium (half MS10; Murashige and Skoog, 1962) comprised 2.2 g/L Murashige and Skoog medium (SIGMA M5519), 10 g/L sucrose, adjusted to pH 5.7 with 1 M KOH, and 8 g/L agar (SIGMA A1296). For the high and low K^+^ media, a stock of growth medium was made up lacking K^+^: the stock consisted of 1.497 mM CaCl_2_, 0.363 mM Ca(H_2_PO_4_)_2_, 10.3 mM NH_4_N0_3_, 0.7506 mM MgSO_4_.7H_2_O, 29.21 mM sucrose, 50 mL/L half-strength MS Vitamins 10× and 500 *µ*L/L half-strength Murashige and Skoog basal salt micronutrients from 1,000× stock. The solution was adjusted to pH 5.7 with 1 M NaOH solution. Different concentrations of K^+^ ions were added to the media using K_2_SO_4_ to achieve final K^+^ concentrations of 2 mM (K^+^-sufficient) and 0.005 mM (K^+^-deprived), and 8 g/L agar (SIGMA A1296) was added. Final [K^+^] of 2 and 0.005 mM were selected following a phenotyping study using a K^+^-concentration gradient from 20 to 0.005 mM (Supplemental Figure S1) and consensus was obtained with published response phenotypes (Kellermeier et al., 2013).

### RNA extraction and RNA-Seq library preparation

For RNA-seq, three biological replicates treatments were used with seedlings sampled at 3 and 30 h after transfer of seedlings to either sufficient or deprived K^+^ media. Whole seedlings (total 100 mg FW) were flash frozen in liquid nitrogen and used to identify changes in gene expression in aerial parts as well as roots. Tissue was ground using a TissueLyser II (QIAGEN^®^, Manchester, UK) before RNA extraction. RNA extraction was carried out using the SIGMA Spectrum™ Plant Total RNA Kit following the manufacturer’s instructions (RT-qPCR analysis) or using a Trizol (TRI Reagent^®^ SIGMA) chloroform extraction method followed by washes described in the SIGMA Spectrum™ Plant Total RNA Kit (RNA-Seq analysis). An on-column DNA digestion was also carried out for all samples (SIGMA). The extracted RNA was analyzed using a Nanodrop 1000 spectrophotometer (ThermoFisher Scientific, Hemel Hempstead, UK). Samples for RNA-Seq were also analyzed on the Agilent 2200 TapeStation, where RNA samples with RNA integration number equivalent (RIN^e^) ˃7.0 were taken forward to library preparation.

RNA-Seq library preparation was completed using the NEBNext^®^ Ultra™ Directional RNA Library Prep Kit for Illumina^®^ protocol for use with NEBNext Poly(A) mRNA Magnetic Isolation Module (NEB #E7490) following the manufacturer’s instructions (NEB, Hitchin, UK). Total RNA of between 100 ng and 1 *µ*g was used. Library quality was assessed using a DNA analysis ScreenTape on the Agilent Technologies 2200 TapeStation. RT-qPCR was then used for sample quantification using NEBNext^®^ Library Quant Kit Quick Protocol Quant kit for Illumina^®^. Samples were diluted to 10 nM. Seven microliters of each 10 nM sample were pooled together and all were run on one lane using the Illumina HiSeq 2500, through the DBS Genomics facility at Durham University.cDNA synthesis for PCR and RT-qPCR was carried out using 5 ng of RNA in a 20 *μ*L reaction mixture. The reactions used SuperScript^®^ III First-Strand Synthesis Supermix (Invitrogen Ltd., Paisley, UK) following the manufacturer’s protocol and primed with Oligo(dT)_20_. The cDNA samples were diluted with sterile distilled water in the ratio of 1:4 before use in PCR and RT-qPCR. PCR amplification with *ACT2* primers (see Supplemental Table S3 for primer sequences) designed over an intron was used to ensure cDNA samples were not contaminated with genomic DNA.

### RT-qPCR

*AT1G13320* was used as a reference gene for all RT-qPCR analyses due to its stable expression profile across a wide range of developmental and environmental conditions (Czechowski et al., 2005), and its consistency across K^+^ concentrations. RT-qPCR reactions were conducted using 2× SensiFAST SYBR^®^ No-ROX Mix and were run on a Rotor-Gene Q Machine (QIAGEN, Hilden, Germany). Expression analysis was conducted using the Rotor-Gene Q Series software version 1.7. Relative normalized levels of transcript of each gene were calculated relative to the reference gene and analyzed by comparative quantification using an assumption-free, linear regression analysis approach (Ramakers et al., 2003). Primer sequences are listed in Supplemental Table S3 or, for DELLA genes, in Supplemental Figure S9.

### Analysis of RNA-Seq data

Results from the Illumina HiSeq 2500 were processed using the following steps. Trimmomatic (Bolger et al., 2014) was used to cut down and remove low-quality reads, TopHat2 (Kim et al., 2013) was used for the alignment of reads against TAIR10 (EnsemblePlants), SAMtools (Li et al., 2009) indexed and sorted the binary sequence alignment files (BAM files) then converted them into readable (SAM) files. HTSeq version 0.6.1 (Anders et al., 2015) was used to estimate gene counts, then EdgeR (Robinson et al., 2010; McCarthy et al., 2012) normalized gene counts and estimated differential expression between sample groups. A *P* ≤0.05, a log-fold change ≥0.5, and FDR < 0.05 were selected to identify DEGs. This *P*-value was used because K^+^-deprivation does not lead to major alterations in transcript abundance (Maathuis et al., 2003; Gierth et al., 2005; Ma et al., 2012). GO enrichment analysis was carried out using the online tool agriGO (Du et al., 2010; Tian et al., 2017), and further analyzed using the online tool REVIGO, which summarizes the list of GO terms and reduces functional redundancies allowing the visualization of the data in easy to interpret formats (Supek et al., 2011).

### Root length analysis

Vertical plates were scanned using a flatbed scanner (Epson Expression 1680Pro, Epson, UK) at resolution 600 dpi. Primary root (PR) length, lateral root (LR) number, and LR length were analyzed from these images using ImageJ (Schneider et al., 2012) with the plugin SmartRoot (Lobet et al., 2011). All LRs were measured when they were long enough to be identified by the analysis software (*ca.* >200 *µ*m). Data from ImageJ were then transferred to Microsoft Excel to produce graphs. Anchor roots (defined as roots emerging from the hypocotyl–root junction; Ingram et al., 2011) were discounted from analysis. The auxin reporter *DR5::GUS* (Sabatini et al., 1999) was used as a marker for early LR development in LR progression analysis. Tissue localization of GUS enzyme activity was performed as described by Topping and Lindsey (1997), and roots were examined using compound light microscopy.

### Compound light microscopy

Histological tissue sections were mounted in chloral hydrate solution (8 g chloral hydrate, 1 mL glycerol, 2 mL water) and examined by compound light microscopy using a Zeiss Axioskop microscope (Carl Zeiss, Cambridge, UK) equipped with a QImaging Retiga-2000r camera (Photometrics, Marlow, UK) and a ×20 objective.

### Confocal scanning laser microscopy

To reveal cell organization, roots were stained in 0.5 *µ*g/mL propidium iodide (PI) solution for 1 min 30 s, then washed for 1 min in sterile distilled water. Roots were then mounted on slides in sdH_2_O, a 1.5-mm cover slip was placed on top, secured by Micropore™ tape and imaged using a Leica SP5 TCS confocal microscope (www.leica-microsystems.com) using either ×40 or ×63 oil immersion objectives. Excitation of fluorophores was performed as follows: GFP at 488 nm using the Argon laser, and PI at 548 nm using the HeNe laser.

### Image analysis

Image J (Schneider et al., 2012) was used for analysis. For analysis of meristem size, the straight-line tool was used to draw a line and measure from the QC to the end of the meristem (defined as the first cell that was twice the length of the immediately preceding cell; González-García et al., 2011). For analysis of *proRGA::RGA::GFP*, the polygon tool was used to draw around the meristem of the LRs and the mean green channel intensity was calculated using the color histogram tool. Background was measured and subtracted from the value.

### Statistical analysis

All statistical analyses were performed in IBM SPSS Statistics for Windows version 22 (Armonk, NY, USA; IBM Corp.). The 0.05 level of significance was used. The one-way analysis of variance and Tukey pairwise comparison *post hoc* test were used to determine significance between the means of ≥3 independent groups. An independent samples *t* test was used to determine significance between the means of two independent groups.

### Accession numbers

SFR6: AT4G04920; JUB1: AT2G43000; CBF1: AT4G25490; ERF5: AT5G47230; ERF6: AT4G17490; RGA: AT2G01570.

## Supplemental data

The following materials are available in the online version of this article.

**Supplemental Figure S1** LR growth on different K^+^ concentrations.

**Supplemental Figure S2** Effect of K^+^-deprivation on LR cells.

**Supplemental Figure S3** Treemap output from REVIGO (Supek et al., 2011) of the genes identified as significantly upregulated after 3h K^+^ starvation following RNA-Seq.

**Supplemental Figure S4** Treemap output from REVIGO (Supek et al., 2011) of the genes identified as significantly downregulated after 3h K^+^ starvation following RNA-Seq.

**Supplemental Figure S5** Treemap output from REVIGO (Supek et al., 2011) of the genes identified as significantly upregulated after 30h K^+^ starvation following RNA-Seq.

**Supplemental Figure S6** Treemap output from REVIGO (Supek et al., 2011) of the genes identified as significantly downregulated after 30 h K^+^ starvation following RNA-Seq.

**Supplemental Figure S7** The effect of auxin supplementation on the LR growth response to K^+^-deprivation.

**Supplemental Figure S8** *JUB1* gene expression and predicted protein interactions.

**Supplemental Figure S9** DELLA gene expression following K^+^ treatment.

**Supplemental Figure S10** Effect of GA on LR meristem activity under K^+^-deprivation.

**Supplemental Figure S11** Proposed model for how K^+^-deprivation affects LR growth through transient regulation of GA and DELLA levels in Arabidopsis Col-0.

**Supplemental Table S1** DEG list 3 and 30 h K^+^-deprivation

**Supplemental Table S2** List of genes both upregulated by K^+^ starvation (either after 3 or 30 h, or both) and also identified by GO analysis as relating to ethylene signaling

**Supplemental Table S3** Primer sequences for RT-qPCR

## Supplementary Material

kiaa093_Supplementary_Data

## Notes

F.M.H., J.F.T., and K.L. designed the experimental work, F.M.H. and K.L. wrote the paper, and F.M.H and MK carried out the experimental work.

## References

[kiaa093-B1] Achard P , Cheng H, De Grauwe L, Decat J, Schoutteten H, Moritz T, Van Der Straeten D, Peng J, Harberd NP (2006) Integration of plant responses to environmentally activated phytohormonal signals. Science 311**:** 91–94 16400150 10.1126/science.1118642

[kiaa093-B2] Achard P , Genschik P (2009) Releasing the brakes of plant growth: how GAs shutdown Della proteins. J Exp Bot 60**:** 1085–1092 19043067 10.1093/jxb/ern301

[kiaa093-B3] Achard P , Gong F, Cheminant S, Alioua M, Hedden P, Genschik P (2008a) The cold-inducible CBF1 factor-dependent signalinng pathway modulates the accumulation of the growth- repressing DELLA proteins via its effect on gibberellin metabolism. Plant Cell 20**:** 2117–2129 18757556 10.1105/tpc.108.058941 PMC2553604

[kiaa093-B4] Achard P , Renou JP, Berthomé R, Harberd NP, Genschik P (2008b) Plant DELLAs restrain growth and promote survival of adversity by reducing the levels of reactive oxygen species. Curr Biol 18**:** 656–660 18450450 10.1016/j.cub.2008.04.034

[kiaa093-B5] Adams F (1971) Soil solution. *In* Carson EW, ed, The Plant Root and its Environment. Charlottesville, VA, University Press of Virginia, pp 441–481

[kiaa093-B6] Anders S , Pyl PT, Huber W (2015) HTSeq-A Python framework to work with high-throughput sequencing data. Bioinformatics 31**:** 166–169 25260700 10.1093/bioinformatics/btu638 PMC4287950

[kiaa093-B7] Armengaud P , Breitling R, Amtmann A (2004) The potassium-dependent transcriptome of Arabidopsis reveals a prominent role of jasmonic acid in nutrient signaling. Plant Physiol 136**:** 2556–2576 15347784 10.1104/pp.104.046482 PMC523322

[kiaa093-B8] Armengaud P , Breitling R, Amtmann A (2010) Coronatine-insensitive 1 (COI1) mediates transcriptional responses of *Arabidopsis thaliana* to external potassium supply. Mol Plant 3**:** 390–405 20339157 10.1093/mp/ssq012 PMC2845782

[kiaa093-B9] Bellini C , Pacurar DI, Perrone I (2014) Adventitious roots and lateral roots: similarities and differences. Ann Rev Plant Biol 65**:** 639–666 24555710 10.1146/annurev-arplant-050213-035645

[kiaa093-B10] Benjamins R , Ampudia CSG, Hooykaas PJJ, Offringa R (2003) PINOID-mediated signaling involves calcium-binding proteins. Plant Physiol 132**:** 1623–1630 12857841 10.1104/pp.103.019943 PMC167099

[kiaa093-B11] Benková E , Michniewicz M, Sauer M, Teichmann T, Seifertová D, Jürgens G, Friml J (2003) Local, efflux-dependent auxin gradients as a common module for plant organ formation. Cell 115**:** 591–602 14651850 10.1016/s0092-8674(03)00924-3

[kiaa093-B13] Bolger AM , Lohse M, Usadel B ( 2014) Trimmomatic: a flexible trimmer for Illumina sequence data. Bioinformatics 30**:** 2114–2120 24695404 10.1093/bioinformatics/btu170 PMC4103590

[kiaa093-B14] Canales J , Moyano TC, Villarroel E, Gutiérrez RA (2014) Systems analysis of transcriptome data provides new hypotheses about *Arabidopsis* root response to nitrate treatments. Front Plant Sci 5**:** 22 24570678 10.3389/fpls.2014.00022 PMC3917222

[kiaa093-B15] Cao SQ , Su L, Fang YJ (2006) Evidence for involvement of jasmonic acid in the induction of leaf senescence by potassium deficiency in *Arabidopsis*. Can J Bot 84**:** 328–333

[kiaa093-B16] Chérel I , Lefoulon C, Boeglin M, Sentenac H (2014) Molecular mechanisms involved in plant adaptation to low K^+^ availability. J Exp Bot 65**:** 833–848 24293613 10.1093/jxb/ert402

[kiaa093-B17] Colebrook EH , Thomas SG, Phillips AL, Hedden P (2014) The role of gibberellin signalling in plant responses to abiotic stress. J Exp Bot 217**:** 67–75 10.1242/jeb.089938 24353205

[kiaa093-B18] Cowling RJ , Kamiya Y, Seto H, Harberd NP (1998) Gibberellin dose-response regulation of *GA4* gene transcript levels in Arabidopsis. Plant Physiol 117**:** 1195–1203 9701576 10.1104/pp.117.4.1195 PMC34884

[kiaa093-B19] Czechowski T , Stitt M, Altmann T, Udvardi MK, Scheible WR (2005) Genome-wide identification and testing of superior reference genes for transcript normalization in Arabidopsis. Plant Physiol 139**:** 5–17 16166256 10.1104/pp.105.063743 PMC1203353

[kiaa093-B20] Dello Ioio R , Linhares FS, Scacchi E, Casamitjana-Martinez E, Heidstra R, Costantino P, Sabatini S **(** 2007) Cytokinins determine Arabidopsis root-meristem size by controlling cell differentiation. Curr Biol 17**:** 678–682 17363254 10.1016/j.cub.2007.02.047

[kiaa093-B21] Dello Ioio R , Nakamura K, Moubayidin L, Perilli S, Taniguchi M, Morita MT, Aoyama T,, Costantino P, Sabatini S (2008) A genetic framework for the control of cell division and differentiation in the root meristem. Science 322**:** 1380–1384 19039136 10.1126/science.1164147

[kiaa093-B22] Desbrosses G , Josefsson C, Rigas S, Hatzopoulos P, Dolan L (2003) *AKT1* and *TRH1* are required during root hair elongation in *Arabidopsis*. J Exp Bot 54**:** 781–788 12554721 10.1093/jxb/erg066

[kiaa093-B23] Du Z , Zhou X, Ling Y, Zhang Z, Su Z (2010) agriGO: a GO analysis toolkit for the agricultural community. Nucl Acids Res 38**:** W64–W70 20435677 10.1093/nar/gkq310 PMC2896167

[kiaa093-B24] Dubois M , Skirycz A, Claeys H, Maleux K, Dhondt S, De Bodt S, Vanden Bossche R, De Milde L, Yoshizumi T, Matsui M (2013) The ETHYLENE RESPONSE FACTOR 6 acts as central regulator of leaf growth under water limiting conditions in *Arabidopsis thaliana*. Plant Physiol 162**:** 319–332 23553636 10.1104/pp.113.216341 PMC3641212

[kiaa093-B25] Dubois M , Van den Broeck L, Claeys H, Van Vlierberghe K, Matsui M, Inzé D (2015) The ETHYLENE RESPONSE FACTORs ERF6 and ERF11 antagonistically regulate mannitol- induced growth inhibition in Arabidopsis. Plant Physiol 169**:** 166–179 25995327 10.1104/pp.15.00335 PMC4577380

[kiaa093-B26] Eliasson L , Bertell G, Bolander E (1989) Inhibitory action of auxin on root elongation not mediated by ethylene. Plant Physiol 91**:** 310–314 16667017 10.1104/pp.91.1.310 PMC1061992

[kiaa093-B27] Fleet CM , Sun TP (2005) A DELLAcate balance: the role of gibberellin in plant morphogenesis. Curr Opin Plant Biol 8**:** 77–85 15653404 10.1016/j.pbi.2004.11.015

[kiaa093-B28] Forzani C , Aichinger E, Sornay E, Willemsen V, Laux T, Dewitte W, Murray JH (2014) WOX5 145 suppresses *CYCLIN D* activity to establish quiescence at the center of the root stem cell niche. Curr Biol 24**:** 1939–1944 25127220 10.1016/j.cub.2014.07.019 PMC4148176

[kiaa093-B29] Fu X , Harberd NP (2003) Auxin promotes *Arabidopsis* root growth by modulating gibberellin response. Nature 421**:** 740–743 12610625 10.1038/nature01387

[kiaa093-B31] Gierth M , Mäser P, Schroeder J (2005) The potassium transporter *AtHAK5* functions in K^+^ deprivation-induced high-affinity Kuptake and AKT1 K^+^ channel contribution to K^+^ uptake kinetics in Arabidopsis roots. Plant Physiol 137**:** 1105–1114 15734909 10.1104/pp.104.057216 PMC1065410

[kiaa093-B33] González-García MP , Vilarrasa-Blasi J, Zhiponova M, Divol F, Mora-García S, Russinova E, Caño-Delgado AI (2011) Brassinosteroids control meristem size by promoting cell cycle progression in Arabidopsis roots. Development 138**:** 849–859 21270057 10.1242/dev.057331

[kiaa093-B34] Gruber BD , Giehl RFH, Friedel S, von Wiren N (2013) Plasticity of the Arabidopsis root system under nutrient deficiencies. Plant Physiol 163**:** 161–179 23852440 10.1104/pp.113.218453 PMC3762638

[kiaa093-B35] Hammer GL , Dong Z, McLean G, Doherty A, Messina C, Schussler J, Zinselmeier C, Paszkiewicz S, Cooper M (2009) Can changes in canopy and/or root system architecture explain historical maize yield trends in the U.S. corn belt? Crop Sci 49**:** 299–312

[kiaa093-B36] Hedden P , Kamiya Y (1997) Gibberellin biosynthesis: enzymes, genes and their regulation. Ann Rev Plant Physiol Plant Mol Biol 48**:** 431–460 15012270 10.1146/annurev.arplant.48.1.431

[kiaa093-B37] Heisler MG , Oh C, Das P, Sieber P, Reddy GV, Long JA, Meyerowitz EM (2005) Patterns of auxin transport and gene expression during primordium development revealed by live imaging of the Arabidopsis inflorescence meristem. Curr Biol 15**:** 1899–1911 16271866 10.1016/j.cub.2005.09.052

[kiaa093-B38] Hentrich M , Böttcher C, Düchting P, Cheng Y, Zhao Y, Berkowitz O, Masle J, Medina J, Pollmann S (2013) The jasmonic acid signaling pathway is linked to auxin homeostasis through the modulation of *YUCCA8* and *YUCCA9* gene expression. Plant J 74**:** 626–637 23425284 10.1111/tpj.12152 PMC3654092

[kiaa093-B39] Ingram P , Dettmer J, Helariutta Y, Malamy JE (2011) Arabidopsis lateral root development3 is essential for early phloem development and function, and hence for normal root system development. Plant J 68**:** 455–467 21749503 10.1111/j.1365-313X.2011.04700.x

[kiaa093-B41] Jia Y , Ding Y, Shi Y, Xhang X, Gong Z, Yang S (2016) The cbfs triple mutants reveal the essential functions of CBFs in cold acclimation and allow the definition of CBF regulons in Arabidopsis. New Phytol 212**:** 345–353 27353960 10.1111/nph.14088

[kiaa093-B42] Jiang C , Gao X, Liao L, Harberd NP, Fu X (2007) Phosphate starvation root architecture and anthocyanin accumulation responses are modulated by the gibberellin-DELLA signaling pathway in Arabidopsis. Plant Physiol 145**:** 1460–1470 17932308 10.1104/pp.107.103788 PMC2151698

[kiaa093-B43] Jung JY , Shin R, Schachtman DP (2009) Ethylene mediates response and tolerance to potassium deprivation in Arabidopsis. Plant Cell 21**:** 607–621 19190240 10.1105/tpc.108.063099 PMC2660615

[kiaa093-B44] Kellermeier F , Chardon F, Amtmann A (2013) Natural variation of Arabidopsis root architecture reveals complementing adaptive strategies to potassium starvation. Plant Physiol 161**:** 1421–1432 23329148 10.1104/pp.112.211144 PMC3585606

[kiaa093-B45] Kim D , Pertea G, Trapnell C, Pimentel H, Kelley R, Salzberg SL (2013) TopHat2: accurate alignment of transcriptomes in the presence of insertions, deletions and gene fusions. Genome Biol 14**:** R36 23618408 10.1186/gb-2013-14-4-r36 PMC4053844

[kiaa093-B46] Kim MJ , Ciani S, Schachtman DP (2010) A peroxidase contributes to ROS production during Arabidopsis root response to potassium deficiency. Molec Plant 3**:** 420–427 20139158 10.1093/mp/ssp121

[kiaa093-B47] Knight H , Mugford SG, Ülker B, Gao D, Thorlby G, Knight MR (2009**)** Identification of SFR6, a key component in cold acclimation acting post-translationally on CBF function. Plant J 58**:** 97–108 19067974 10.1111/j.1365-313X.2008.03763.x

[kiaa093-B48] Knight H , Veale EL, Warren GJ, Knight MR (1999) The *sfr6* mutation in Arabidopsis suppresses low-temperature induction of genes dependent on the CRT DRE sequence motif. Plant Cell 11**:** 875–886 10330472 10.1105/tpc.11.5.875 PMC144218

[kiaa093-B49] Lavenus J , Goh T, Roberts I, Guyomarch S, Lucas M, De Smet I, Fukaki H, Beeckman T, Bennett M, Laplaze L (2013) Lateral root development in Arabidopsis: fifty shades of auxin. Trends Plant Sci 18**:** 1360–1385 10.1016/j.tplants.2013.04.006 23701908

[kiaa093-B50] Leigh RA , Jones RGW (1984) A hypothesis relating critical potassium concentrations for growth to the distribution and functions of this ion in the plant cell. New Phytol 97**:** 1–13

[kiaa093-B51] Lewis DR , Negi S, Sukumar P, Muday GK **(** 2011) Ethylene inhibits lateral root development, increases IAA transport and expression of PIN3 and PIN7 auxin efflux carriers. Development 138**:** 3485–3495 21771812 10.1242/dev.065102

[kiaa093-B52] Li H , Handsaker B, Wysoker A, Fennell T, Ruan J, Homer N, Marth G, Abecasis G, Durbin R (2009) The sequence alignment/map format and SAM tools. Bioinformatics 25**:** 2078–2079 19505943 10.1093/bioinformatics/btp352 PMC2723002

[kiaa093-B53] Li L , Hou X, Tsuge T, Ding M, Aoyama T, Oka A, Gu H, Zhao Y, Qu LJ (2008) The possible action mechanisms of indole-3-acetic acid methyl ester in Arabidopsis. Plant Cell Rep 27**:** 575–584 17926040 10.1007/s00299-007-0458-9

[kiaa093-B54] Liu Y , Donne E, Lombi E., Li RY, Wu ZC, Zhao FJ, Wu P (2013) Assessing the contributions of lateral roots to element uptake in rice using an auxin-related lateral root mutant. Plant Soil 372**:** 125–136

[kiaa093-B55] Lobet G , Pagès L, Draye X (2011) A novel image-analysis toolbox enabling quantitative analysis of root system architecture. Plant Physiol 157**:** 29–39 21771915 10.1104/pp.111.179895 PMC3165877

[kiaa093-B56] López-Bucio J , Cruz-Ramírez A, Herrera-Estrella L (2003) The role of nutrient availability in regulating root architecture. Curr Opin Plant Biol 6**:** 280–287 12753979 10.1016/s1369-5266(03)00035-9

[kiaa093-B57] Ma TL , Wu WH, Wang Y (2012) Transcriptome analysis of rice root responses to potassium deficiency. BMC Plant Biol 12**:** 161 22963580 10.1186/1471-2229-12-161 PMC3489729

[kiaa093-B58] Maathuis FJM, , Filatov V, Herzyk P, Krijger GC, Axelsen KB, Chen S, Green BJ, Li Y, Madagan KL, Sánchez-Fernández R, Forde BG et al (2003) Transcriptome analysis of root transporters reveals participation of multiple gene families in the response to cation stress. Plant J 35**:** 675–692 12969422 10.1046/j.1365-313x.2003.01839.x

[kiaa093-B59] Maeght JL , Rewald B, Pierret A (2013) How to study deep roots—and why it matters. Front Plant Sci 4**:** 299 23964281 10.3389/fpls.2013.00299 PMC3741475

[kiaa093-B60] Magome H , Yamaguchi S, Hanada A, Kamiya Y, Oda K (2008) The DDF1 transcriptional activator upregulates expression of a gibberellin-deactivating gene, *GA2ox7*, under high-salinity stress in Arabidopsis. Plant J 56**:** 613–626 18643985 10.1111/j.1365-313X.2008.03627.x

[kiaa093-B61] Malamy JE , Benfey PN (1997) Organization and cell differentiation in lateral roots of *Arabidopsis thaliana*. Development 124**:** 33–44 9006065 10.1242/dev.124.1.33

[kiaa093-B62] Mandadi KK , Misra A, Ren S, McKnight TD (2009) BT2, a BTB protein, mediates multiple responses to nutrients, stresses, and hormones in Arabidopsis. Plant Physiol 150**:** 1930–1939 19525324 10.1104/pp.109.139220 PMC2719139

[kiaa093-B63] Maruyama K , Sakuma Y, Kasuga M, Ito Y, Seki M, Goda H, Shimada Y, Yoshida S, Shinozaki K, Yamaguchi-Shinozaki K **(** 2004 **)** Identification of cold-inducible downstream genes of the Arabidopsis DREB1A/CBF3 transcriptional factor using two microarray systems. Plant J 38**:** 982–993 15165189 10.1111/j.1365-313X.2004.02100.x

[kiaa093-B64] McCarthy DJ , Chen Y, Smyth GK (2012) Differential expression analysis of multifactor RNA-Seq experiments with respect to biological variation. Nucl Acids Res 40**:** 4288–4297 22287627 10.1093/nar/gks042 PMC3378882

[kiaa093-B65] Moffat CS , Ingle RA, Wathugala DL, Saunders NJ, Knight H, Knight MR (2012) ERF5 and ERF6 play redundant roles as positive regulators of JA/Et-mediated defense against *Botrytis cinerea* in Arabidopsis. PLoS One 7**:** e35995 22563431 10.1371/journal.pone.0035995 PMC3338558

[kiaa093-B66] Moore S , Zhang X, Mudge A, Rowe JH, Topping JF, Liu J, Lindsey K **(** 2015) Spatiotemporal modelling of hormonal crosstalk explains the level and patterning of hormones and gene expression in *Arabidopsis thaliana* wild-type and mutant roots. New Phytol 207**:** 1110–1122 25906686 10.1111/nph.13421 PMC4539600

[kiaa093-B67] Moubayidin L , Perilli S, Dello Ioio R, Di Mambro R, Costantino P, Sabatini S (2010) The rate of cell differentiation controls the *Arabidopsis* root meristem growth phase. Curr Biol 20**:** 1138–1143 20605455 10.1016/j.cub.2010.05.035

[kiaa093-B68] Murashige T , Skoog F (1962) A revised medium for rapid growth and bioassays with tobacco tissue cultures. Physiol Plant 15**:** 473–497

[kiaa093-B69] Nam YJ , Tran LS., Kojima M, Sakakibara H, Nishiyama R, Shin R (2012) Regulatory roles of cytokinins and cytokinin signaling in response to potassium deficiency in *Arabidopsis*. PLoS One 7**:** e47797 23112848 10.1371/journal.pone.0047797 PMC3480408

[kiaa093-B71] Novillo F , Medina J, Salinas J (2007) *Arabidopsis* CBF1 and CBF3 have a different function than CBF2 in cold acclimation and define different gene classes in the CBF regulon. Proc Natl Acad Sci USA 104**:** 21002–21007 18093929 10.1073/pnas.0705639105 PMC2409256

[kiaa093-B72] Peng J , Carol P, Richards DE, Kin KE, Cowlin RJ, Murphy GP, Harberd NP (1997) The Arabidopsis *GAI* gene defines a signaling pathway that negatively regulates gibberellin responses. Genes Devel 11**:** 3194–3205 9389651 10.1101/gad.11.23.3194 PMC316750

[kiaa093-B73] Peng J , Richards DE, Hartley NM, Murphy GP, Devos KM, Flintham JE, Beales J, Fish LJ,, Worland AJ, Pelica F et al **(** 1999) ‘ Green revolution’ genes encode mutant gibberellin response modulators. Nature 400**:** 256–261 10421366 10.1038/22307

[kiaa093-B74] Perrot-Rechenmann C (2010) Cellular responses to auxin: division versus expansion. Cold Spring Harbor Persp Biol 2**:** a001446 10.1101/cshperspect.a001446 PMC2857164 20452959

[kiaa093-B75] Pitts RJ , Cernac A, Estelle M (1998) Auxin and ethylene promote root hair elongation in Arabidopsis. Plant J 16**:** 553–560 10036773 10.1046/j.1365-313x.1998.00321.x

[kiaa093-B76] Prajapati K , Modi HA (2012) The importance of potassium in plant growth – a review. Ind J Plant Sci 1**:** 2319–382402

[kiaa093-B77] Qin G , Gu H, Zhao Y, Ma Z, Shi G, Yang Y, Pichersky E, Chen H, Liu M, Chen Z et al (2005) An indole-3-acetic acid carboxyl methyltransferase regulates Arabidopsis leaf development. Plant Cell 17**:** 2693–2704 16169896 10.1105/tpc.105.034959 PMC1242266

[kiaa093-B78] Ramakers C , Ruijter JM, Deprez RHL, Moorman AFM (2003) Assumption-free analysis of quantitative real-time polymerase chain reaction (PCR) data. Neurosci Lett 339**:** 62–66 12618301 10.1016/s0304-3940(02)01423-4

[kiaa093-B79] Rieu I , Eriksson S, Powers SJ, Gong F, Griffiths J, Woolley L, Benlloch R, Nilsson O, Thomas SG, Hedden P et al (2008) Genetic analysis reveals that C19-GA 2-oxidation is a major gibberellin inactivation pathway in *Arabidopsis*. Plant Cell 20**:** 2420–2436 18805991 10.1105/tpc.108.058818 PMC2570722

[kiaa093-B80] Rigas S , Ditengou FA, Ljung K, Daras G, Tietz O, Palme K, Hatzopoulos P ( 2013) Root gravitropism and root hair development constitute coupled developmental responses regulated by auxin homeostasis in the Arabidopsis root apex. New Phytol 197**:** 1130–1141 23252740 10.1111/nph.12092

[kiaa093-B81] Robinson MD , McCarthy DJ, Smyth GK (2010) edgeR: a bioconductor package for differential expression analysis of digital gene expression data. Bioinformatics 26**:** 139–140 19910308 10.1093/bioinformatics/btp616 PMC2796818

[kiaa093-B82] Rowe JH , Topping JF., Liu J, Lindsey K (2016) Abscisic acid regulates root growth under osmotic stress conditions via an interacting hormonal network with cytokinin, ethylene and auxin. New Phytol 211**:** 225–239 26889752 10.1111/nph.13882 PMC4982081

[kiaa093-B83] Sabatini S , Beis D, Wolkenfelt H, Murfett J, Guilfoyle T, Malamy J., Benfey P, Leyser O, Bechtold N, Weisbeek P et al (1999) An auxin-dependent distal organizer of pattern and polarity in the Arabidopsis root. Cell 99**:** 463–472 10589675 10.1016/s0092-8674(00)81535-4

[kiaa093-B84] Sabatini S , Heidstra R, Wildwater M, Scheres B (2003) SCARECROW is involved in positioning the stem cell niche in the Arabidopsis root meristem. Genes Dev 17**:** 354–358 12569126 10.1101/gad.252503 PMC195985

[kiaa093-B85] Sarkar AK , Luijten M, Miyashima S, Lenhard M, Hashimoto T, Nakajima K, Scheres B, Heidstra R, Laux T (2007) Conserved factors regulate signalling in *Arabidopsis thaliana* shoot and root stem cell organizers. Nature 446**:** 811–814 17429400 10.1038/nature05703

[kiaa093-B86] Schneider CA , Rasband WS, Eliceiri KW (2012) NIH image to image J: 25 years of image analysis. Nat Meth 9**:** 671–675 10.1038/nmeth.2089 PMC5554542 22930834

[kiaa093-B87] Schnittger A , Schoebinger U, Stierhof YD, Huelskamp M (2002) Ectopic B-type cyclin expression induces mitotic cycles in endoreduplicating *Arabidopsis* trichomes. Curr Biol 12**:** 415–420 11882294 10.1016/s0960-9822(02)00693-0

[kiaa093-B88] Seki M , Narusaka M, Ishida J, Nanjo T, Fujita M, Oono Y, Kamiya A, Nakajima M, Enju A, Sakurai T, et al (2002) Monitoring the expression profiles of 7000 Arabidopsis genes under drought, cold and high-salinity stresses using a full-length cDNA microarray. Plant J 31**:** 279–292 12164808 10.1046/j.1365-313x.2002.01359.x

[kiaa093-B89] Shahnejat-Bushehri S , Tarkowska D, Sakuraba Y, Balazadeh S **(** 2016) Arabidopsis NAC transcription factor JUB1 regulates GA/BR metabolism and signalling. Nat Plants 2**:** 16013 27249348 10.1038/nplants.2016.13

[kiaa093-B90] Shahnejat-Bushehri S , Allu AD, Mehterov N, Thirumalaikumar VP, Alseekh S, Fernie AR, Mueller-Roeber B, Balazadeh S (2017) Arabidopsis NAC transcription factor JUNGBRUNNEN1 exerts conserved control over gibberellin and brassinosteroid metabolism and signaling genes in tomato. Frontiers Plant Sci 8**:** 214 10.3389/fpls.2017.00214 PMC5339236 28326087

[kiaa093-B91] Shahzad Z , Eaglesfield R, Carr C, Amtmann A (2020) Cryptic variation in RNA-directed DNA-methylation controls lateral root development when auxin signaling is perturbed. Nat Comms 11**:** 218 10.1038/s41467-019-13927-3 PMC6954204 31924796

[kiaa093-B92] Shin R , Burc AY, Huppert KA, Tiwari SB, Murphy AS, Guilfoyle TJ, Schachtman DP (2007) The Arabidopsis transcription factor MYB77 modulates auxin signal transduction. Plant Cell 19**:** 2440–2453 17675404 10.1105/tpc.107.050963 PMC2002618

[kiaa093-B93] Shin R , Schachtman DP (2004) Hydrogen peroxide mediates plant root cell response to nutrient deprivation. Proc Nat Acad Sci USA 101**:** 8827–8832 15173595 10.1073/pnas.0401707101 PMC423280

[kiaa093-B94] Short E , Leighton M, Imriz G, Liu D, Cope-Selby N, Hetherington F, Smertenko A, Hussey PJ, Topping JF, Lindsey K (2018) Epidermal expression of a sterol biosynthesis gene regulates root growth by a non-cell-autonomous mechanism in *Arabidopsis*. Development 145**:** 160572 10.1242/dev.160572 PMC6001376 29695610

[kiaa093-B95] Silverstone AL , Jung HS, Dill A, Kawaide H, Kamiya Y, Sun TP (2001) Repressing a repressor: gibberellin-induced rapid reduction of the RGA protein in Arabidopsis. Plant Cell 13**:** 1555–1566 11449051 10.1105/TPC.010047 PMC139546

[kiaa093-B98] Sun TP , Gubler F **(** 2004) Molecular mechanism of gibberellin signalling in plants. Ann Rev Plant Biol 55**:** 197–223 15377219 10.1146/annurev.arplant.55.031903.141753

[kiaa093-B99] Supek F , Bošnjak M, Škunca N, Šmuc T **(** 2011) Revigo summarizes and visualizes long lists of gene ontology terms. PLoS One 6**:** e21800 21789182 10.1371/journal.pone.0021800 PMC3138752

[kiaa093-B100] Thirumalaikumar VP , Devkar V, Mehterov N, Ali S, Ozgur R, Turkan I, Mueller-Roeber B, Balazadeh S (2018) NAC transcription factor JUNGBRUNNEN1 enhances drought teolerance in tomato. Plant J 16**:** 354–366 10.1111/pbi.12776 PMC5787828 28640975

[kiaa093-B101] Thomas SG , Phillips AL, Hedden P (1999) Molecular cloning and functional expression of gibberellin 2- oxidases, multifunctional enzymes involved in gibberellin deactivation. Proc Nat Acad Sci USA 96**:** 4698–4703 10200325 10.1073/pnas.96.8.4698 PMC16395

[kiaa093-B102] Tian T , Liu Y, Yan H, You Q, Yi X, Du Z, Xu W, Su Z (2017) agriGO v2.0: a GO analysis toolkit for the agricultural community, 2017 update. Nucl Acids Res 45: W122–W129 28472432 10.1093/nar/gkx382 PMC5793732

[kiaa093-B103] Topping JF , Lindsey K (1997) Promoter trap markers differentiate structural and positional components of polar development in Arabidopsis. Plant Cell 9**:** 1713–1725 9368412 10.1105/tpc.9.10.1713 PMC157016

[kiaa093-B104] Vicente-Agullo F , Rigas S, Desbrosses G, Dolan L, Hatzopoulos P, Grabov A (2004) Potassium carrier TRH1 is required for auxin transport in *Arabidopsis* roots. Plant J 40**:** 523–535 15500468 10.1111/j.1365-313X.2004.02230.x

[kiaa093-B105] Wang M , Zheng Q, Shen Q, Guo S (2013) The critical role of potassium in plant stress response. Int J Molec Sci 14**:** 7370–7390 23549270 10.3390/ijms14047370 PMC3645691

[kiaa093-B106] Willige BC , Isono E, Richter R, Zourelidou M, Schwechheimer C (2011) Gibberellin regulates PIN-FORMED abundance and is required for auxin transport-dependent growth abd development in *Arabidopsis thaliana*. Plant Cell 23**:** 2184–2195 21642547 10.1105/tpc.111.086355 PMC3160035

[kiaa093-B107] Wu A , Allu AD, Garapati P, Siddiqui H, Dortay H, Zanor MI, Asensi-Fabado MA, Munne-Bosch S, Antonio C, Tohge T, et al (2012) JUNGBRUNNEN1, a reactive oxygen species-responsive NAC transcription factor, regulates longevity in Arabidopsis. Plant Cell 24**:** 482–506 22345491 10.1105/tpc.111.090894 PMC3315228

[kiaa093-B108] Xu YL , Li L, Gag DA, Zeevaart JA (1999) Feedback regulation of *GA5* expression and metabolic engineering of gibberellin levels in Arabidopsis. Plant Cell 11**:** 927–936 10330476 10.1105/tpc.11.5.927 PMC144230

[kiaa093-B109] Zentella R , Zhang ZL, Park M, Thomas SG, Endo A, Murase K, Fleet M., Jikumaru Y, Nambara E, Kamiya Y, et al. (2007) Global analysis of DELLA direct targets in early gibberellin signaling in Arabidopsis. Plant Cell 19**:** 3037–3057 17933900 10.1105/tpc.107.054999 PMC2174696

[kiaa093-B110] Zhang S , Yang C, Peng J, Sun S, Wang X (2009) GASA5, a regulator of flowering time and stem growth in *Arabnidopsis thaliana*. Plant Mol Biol 69**:** 745–759 19190987 10.1007/s11103-009-9452-7

[kiaa093-B111] Zhao Y , Hull AK, Gupta NR, Goss KA, Alonso J, Ecker JR, Normanly J, Chory J, Celenza JL (2002) Trp-dependent auxin biosynthesis in Arabidopsis: involvement of cytochrome P450s CYP79B2 and CYP79B3. Genes Dev 16**:** 3100–3112 12464638 10.1101/gad.1035402 PMC187496

[kiaa093-B112] Zhou M , Chen H, Wei D, Ma H, Lin J (2017) Arabidopsis CBF3 and DELLAs positively regulate each other in response to low temperature. Sci Rep 7**:** 39819 28051152 10.1038/srep39819 PMC5209670

