# Supplementary material for: Gibberellin signaling mediates lateral root inhibition in response to K+-deprivation
Source: Plant Physiol. 2021 Jan 6;185(3):1198–215. doi: 10.1093/plphys/kiaa093 (PMC8133588; doi:10.1093/plphys/kiaa093)
Supplement: kiaa093_Supplementary_Data [file kiaa093_supplementary_data.zip › pp.01250.2020-s02.pdf]

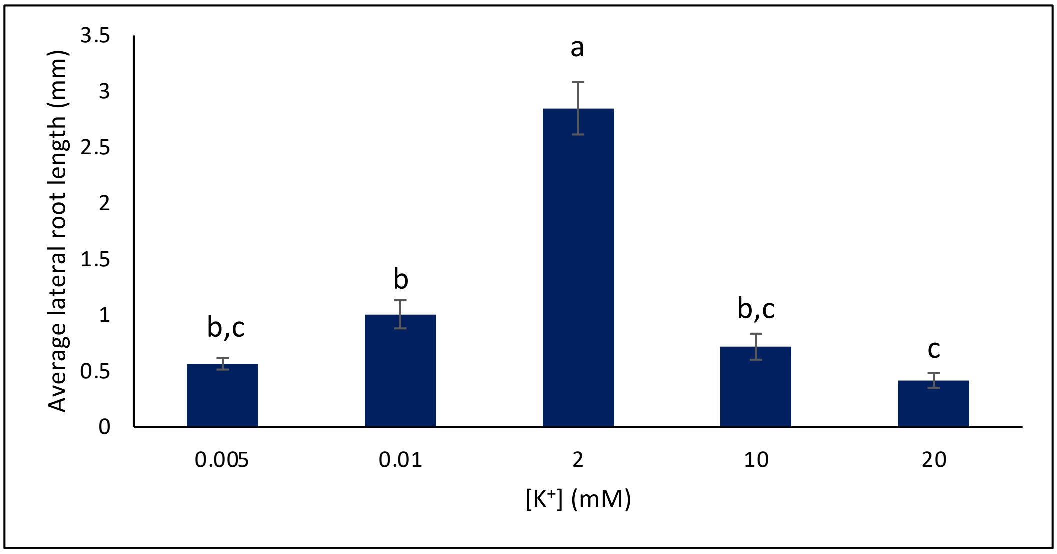

Supplemental Figure S1. Lateral root growth on different K^+ concentrations. Average lateral root length of wild type accession Col-0 seedlings grown for 4 d on ½ MS10 agar plates followed by 8 d on vertical agar plates supplemented with either 0.005 mM, 0.01 mM, 2 mM, 10 mM or 20 mM K^+. Values are means ± SE of at least 23 individual seedlings per treatment. Letters indicate significance with a Tukey Pairwise comparison, $p < 0.05$.

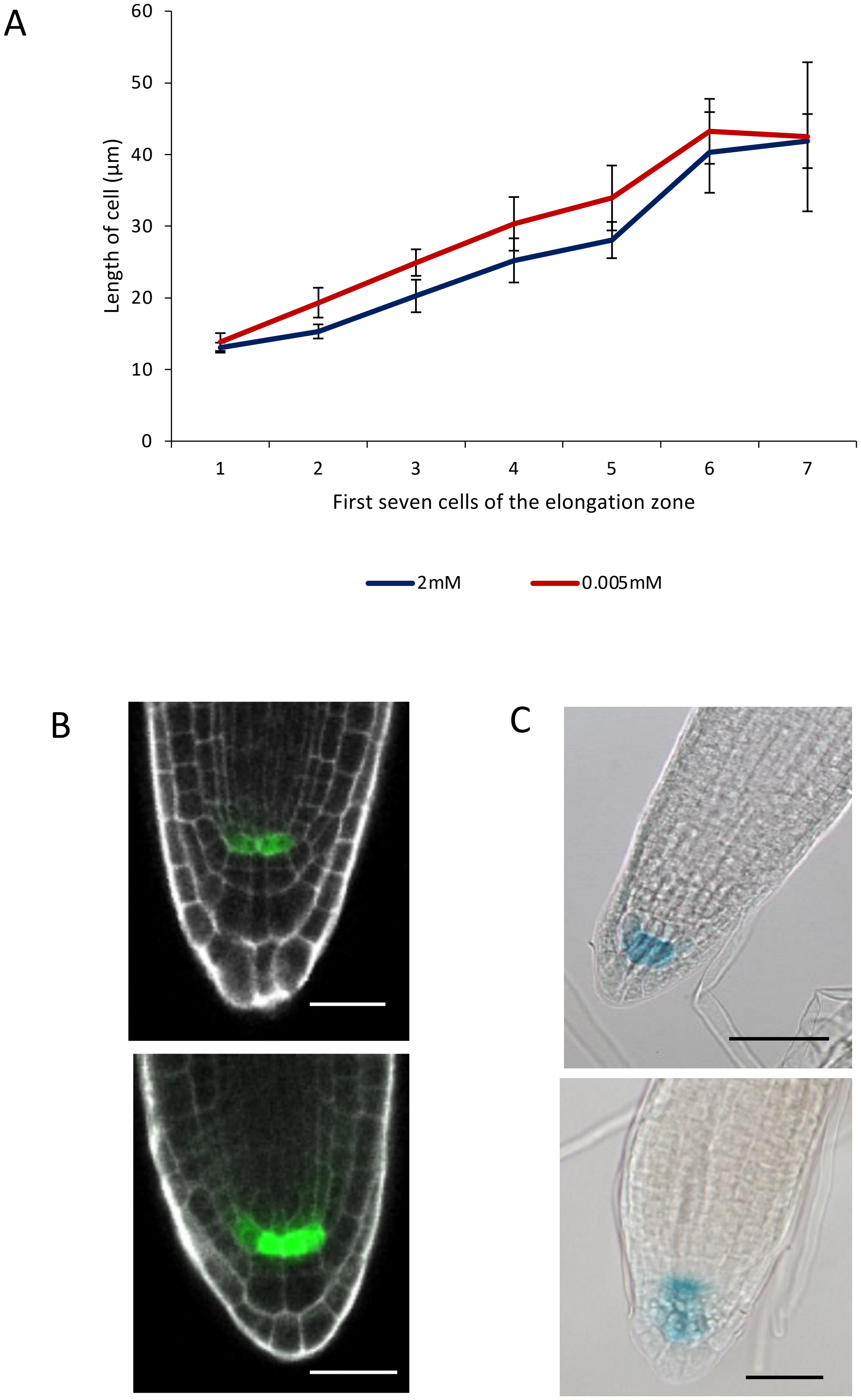

Supplemental Figure S2. Effect of K^+-deprivation on lateral root (LR) cells. A) Length of the first seven cells of the elongation zone of lateral roots grown for 4 d ½ MS10 followed by 8 d on 2 mM or 0.005 mM [K^+]. Measurementstaken from at least 6 different seedlings, $n \geq 11$ for all apart from 7, where n= 6. Values are means ± SE. Independent samples t test found no significance between [K^+] at any of the cells ($p < 0.05$). 1 ($p = 0.597$), 2 (p =0.1), 3 ($p = 0.121$), 4 (p =0.306), 5 (p =0.268), 6 ($p = 0.695$), 7 (p =0.953). (B) *WOX5::GFP* expression in LRs over 200 µm, [K^+] 2 mM (upper) and 0.005 mM (lower); scale bars = 50 µm. (C) *QC25::GUS* expression in LR tips when grown on high (upper panel) and low (lower panel) [K^+]; scale bars = 100 µM.

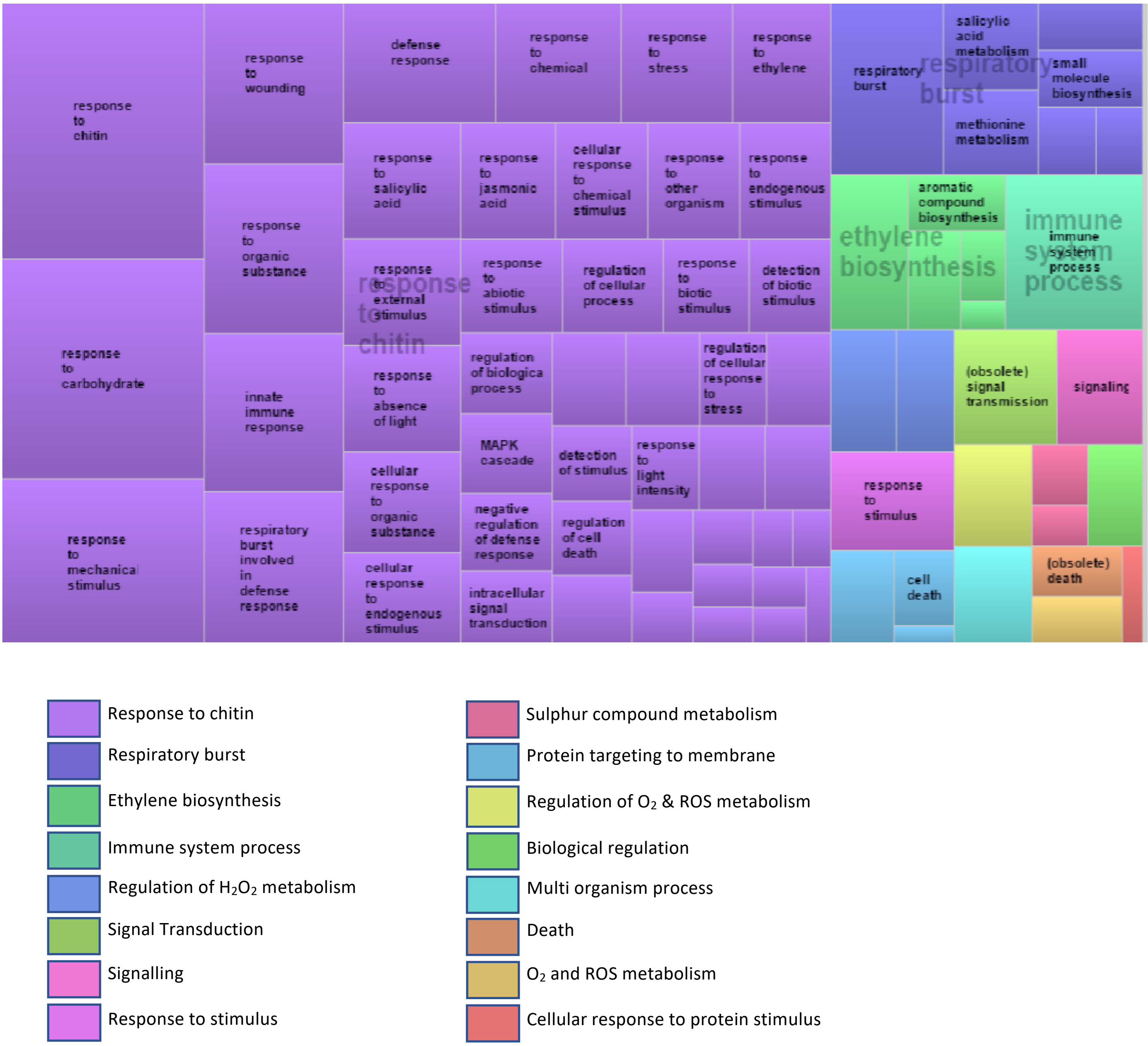

Supplemental Figure S3. Treemap output from REVIGO (Supek et al. 2011) of the genes identified as significantly upregulated after 3h K^+ starvation, following RNA-Seq. P value ≤ 0.05 and a log fold change (log_2FC) ≥ 0.5. Each rectangle represents a GO term cluster and each colour represents a supercluster of related clusters. The superclusters are listed in the key below the treemap. Sizes of rectangle reflect the $-log_{10}$ p-value of each cluster.

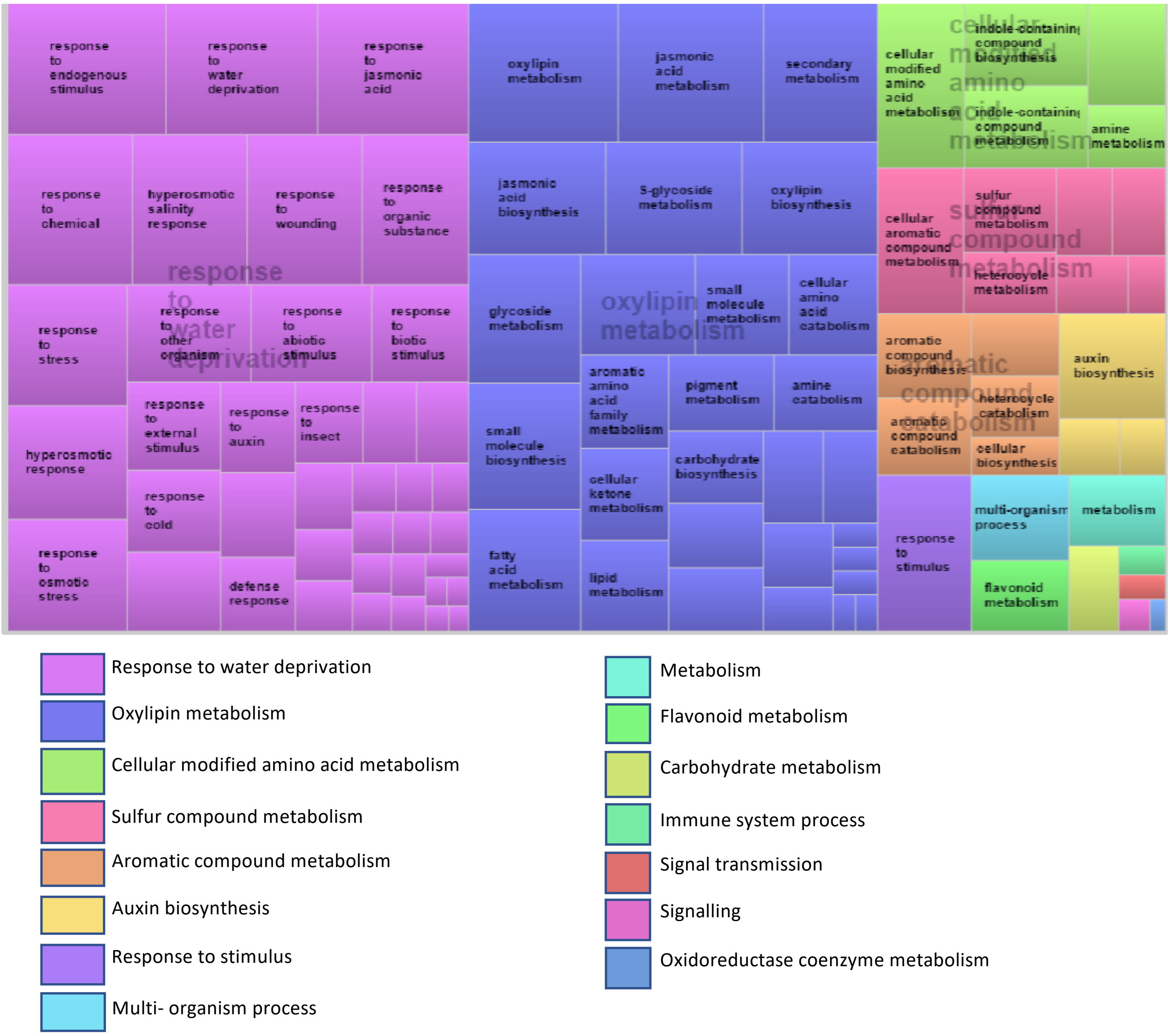

Supplemental Figure S4. Treemap output from REVIGO (Supek et al, 2011) of the genes identified as significantly downregulated after 3hrs K^+ starvation, following RNA-Seq. P value ≤ 0.05 and a log fold change ($\log_2 FC$) ≤ -0.5. Each rectangle represents a GO term cluster and each colour represents a supercluster of related clusters. The superclusters are listed in the key below the treemap. Sizes of rectangle reflect the $-\log_{10}$ p-value of each cluster.

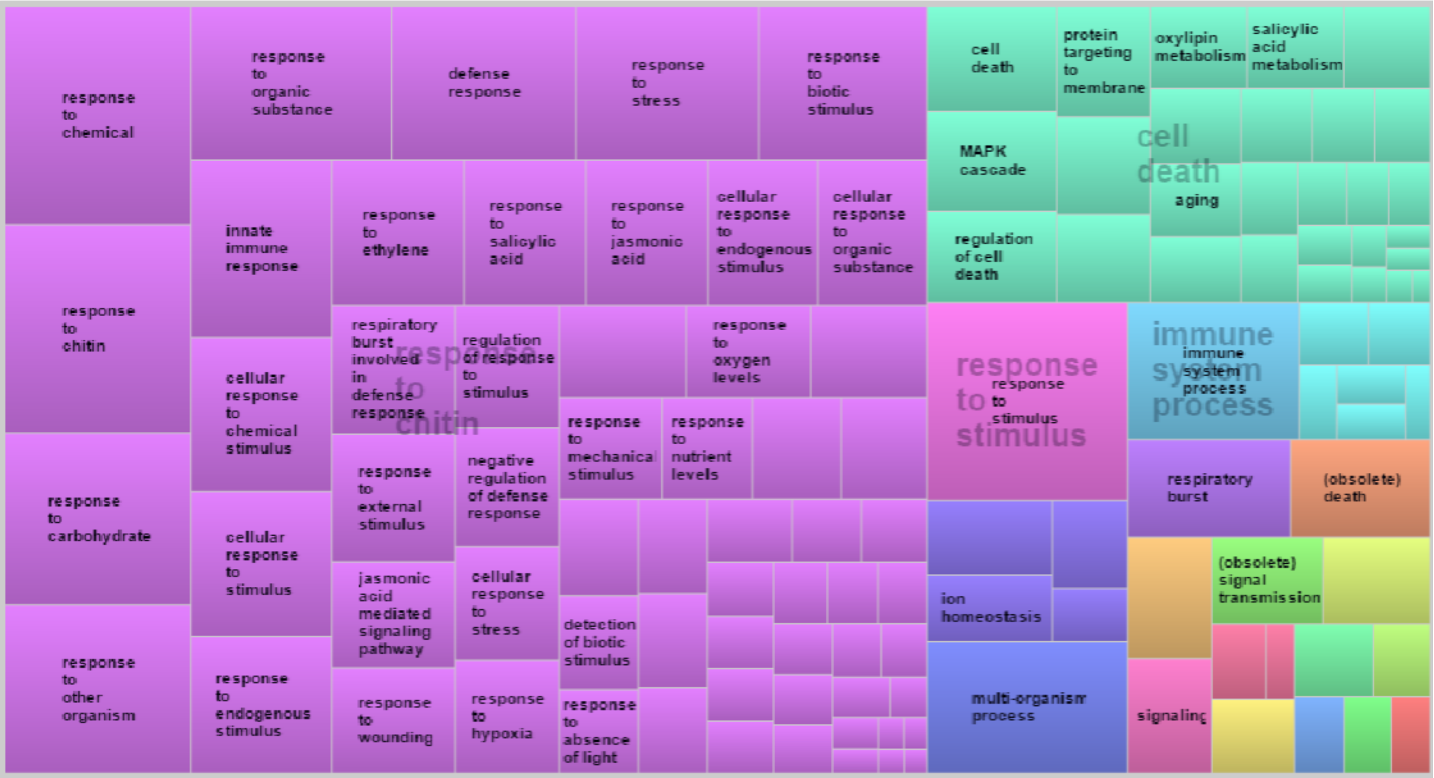

- Response to chitin
- Cell death
- Response to stimulus
- Immune system process
- Ethylene biosynthesis
- Regulation of H_2O_2 metabolism
- Multi-organism process
- Respiratory burst
- Death
- Di-, tri-valent inorganic cation homeostasis
- Signal transmission (Obsolete)
- Regulation of O_2 & ROS metabolism
- Signalling
- Sulfur compound metabolism
- Cell communication
- Senescence
- O_2 & ROS metabolism
- Localization
- Biological regulation
- Cellular di-, tri-valent inorganic cation homeostasis

Supplemental Figure S5. Treemap output from REVIGO (Supek et al. 2011) of the genes identified as significantly upregulated after 30 h K^+ starvation, following RNA-Seq. P value ≤ 0.05 and a log fold change (log_2FC) ≥0.5. Each rectangle represents a GO term cluster and each colour represents a supercluster of related clusters. The superclusters are listed in the key below the treemap. Sizes of rectangle reflect the $–log_{10}$ p-value of each cluster.

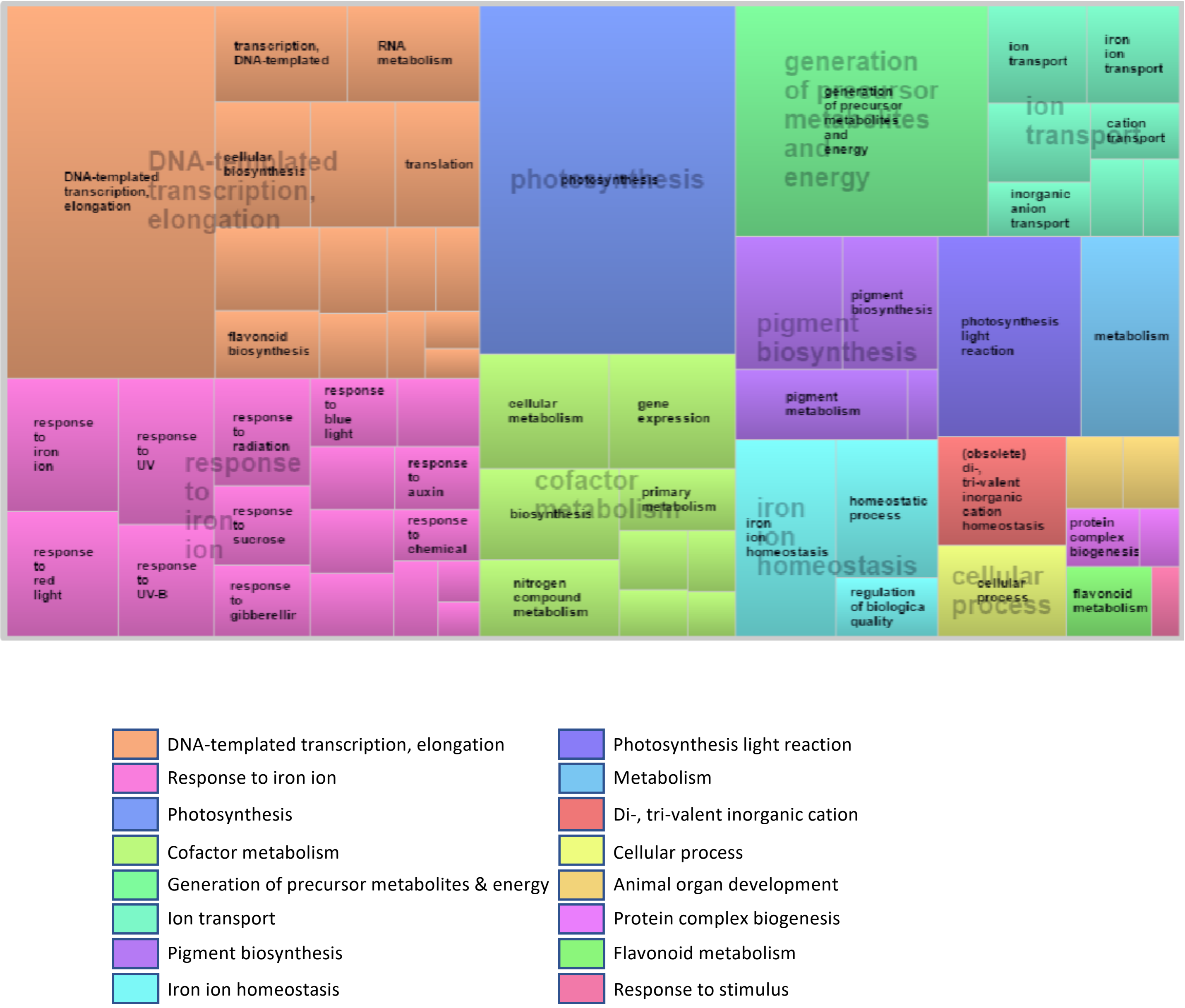

Supplemental Figure S6.
Treemap output from REVIGO (Supek et al. 2011) of the genes identified as significantly downregulated after 30 h K^+ starvation, following RNA-Seq. P value ≤ 0.05 and a log fold change (log_2FC) ≤ -0.5. Each rectangle represents a GO term cluster and each colour represents a supercluster of related clusters. The superclusters are listed in the key below the treemap.
Sizes of rectangle reflect the $–log_{10}$ p-value of each cluster.

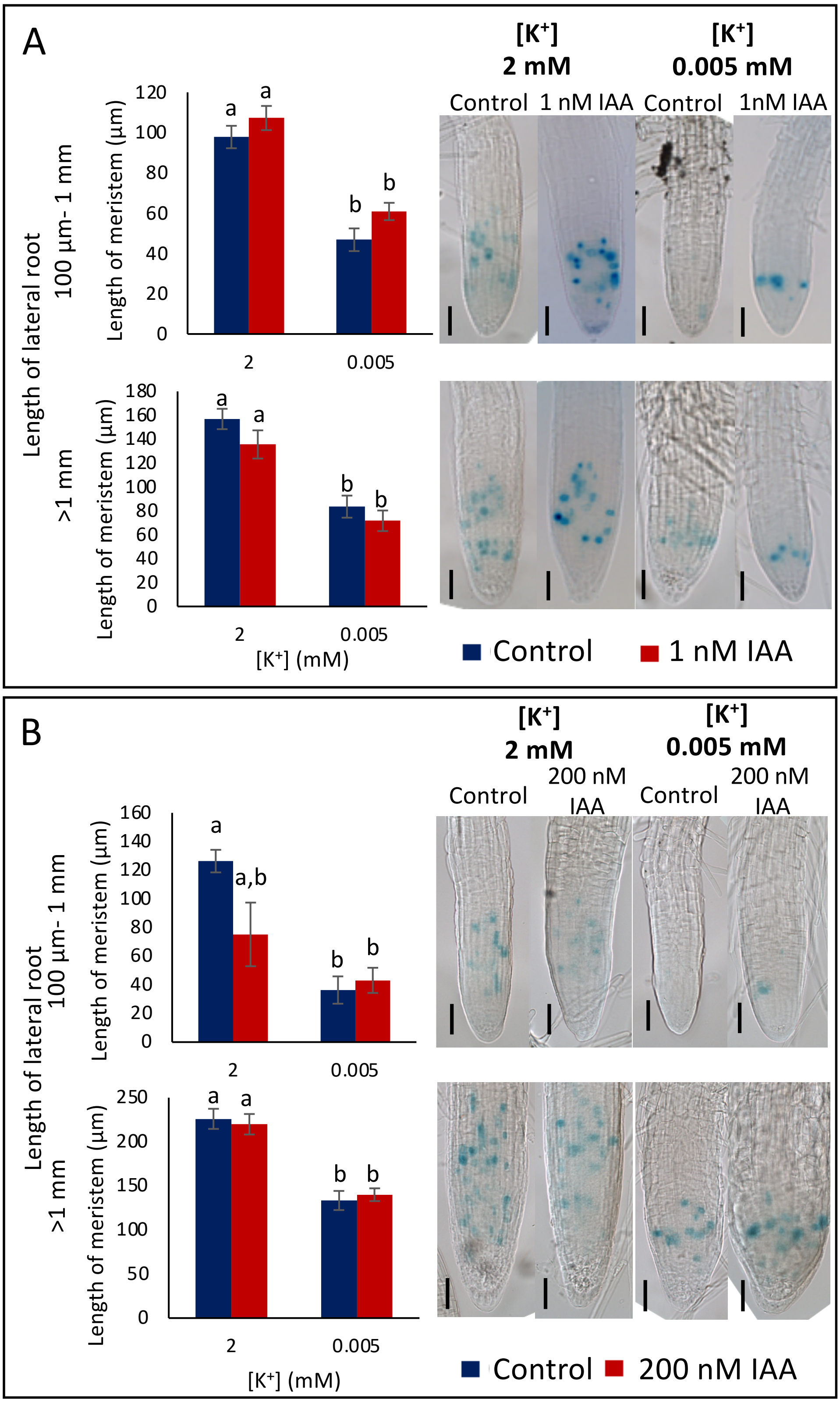

Supplemental Figure S7. The effect of auxin supplementation on the lateral root (LR) growth response to K^+-deprivation.

Typical GUS staining pattern of *CYCB1;2::GUS*; the staining shows a reduced area of cell division in LRs grown on 0.005 mM [K^+] compared to 2 mM. Scale bars = 50 µM. Mean length of meristem measured as the length of area with dividing cells (stained blue in *CYCB1;2::GUS* line). Medium supplemented with either 1 nM IAA (A), or 200 nM IAA (B) for 8 d. Analysis carried out on seedlings 12 DAG. Values are mean measurements taken from at least 6 individual seedlings per treatment ± SE, Letters indicate significance with a Tukey Pairwise comparison, $p < 0.05$.

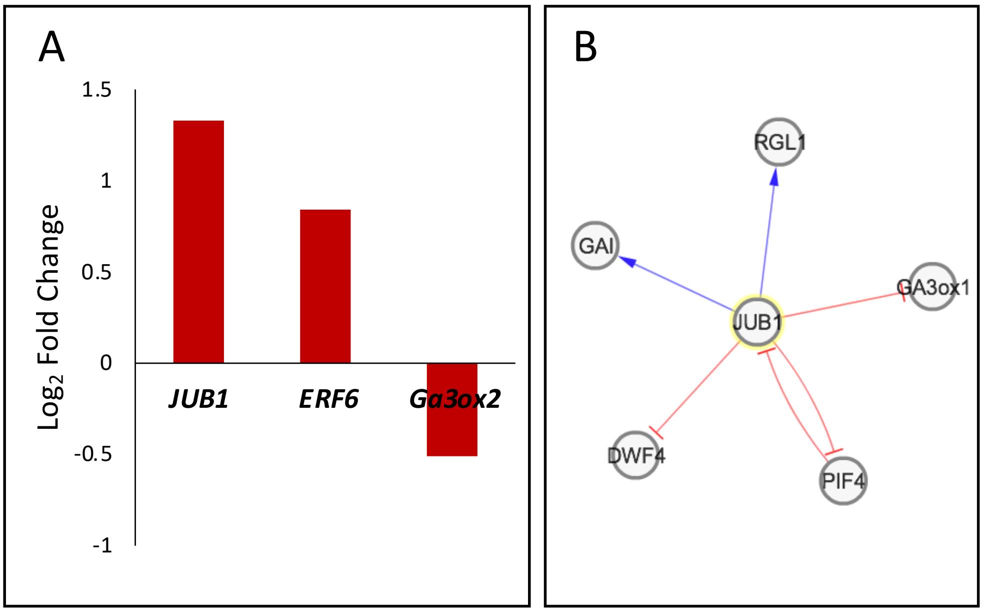

Supplemental Figure S8. *JUB1* gene expression and predicted protein interactions. (A) Changes in expression of *ERF6, JUB1* and *GA3ox2* after 30 h K^+ treatment. (B) Output from Agris AtTFDB Arabidopsis transcription factor database (Davuluri *et al.,* 2003; Yilmaz *et al.*, 2011) identifying interactions between JUB1 (NAC42) (AT2G43000) and other proteins. *JUB1* is a H_2O_2 induced NAC transcription factor gene upregulated by 1.33$\log_2$fc after 30 h K^+-deprivation.

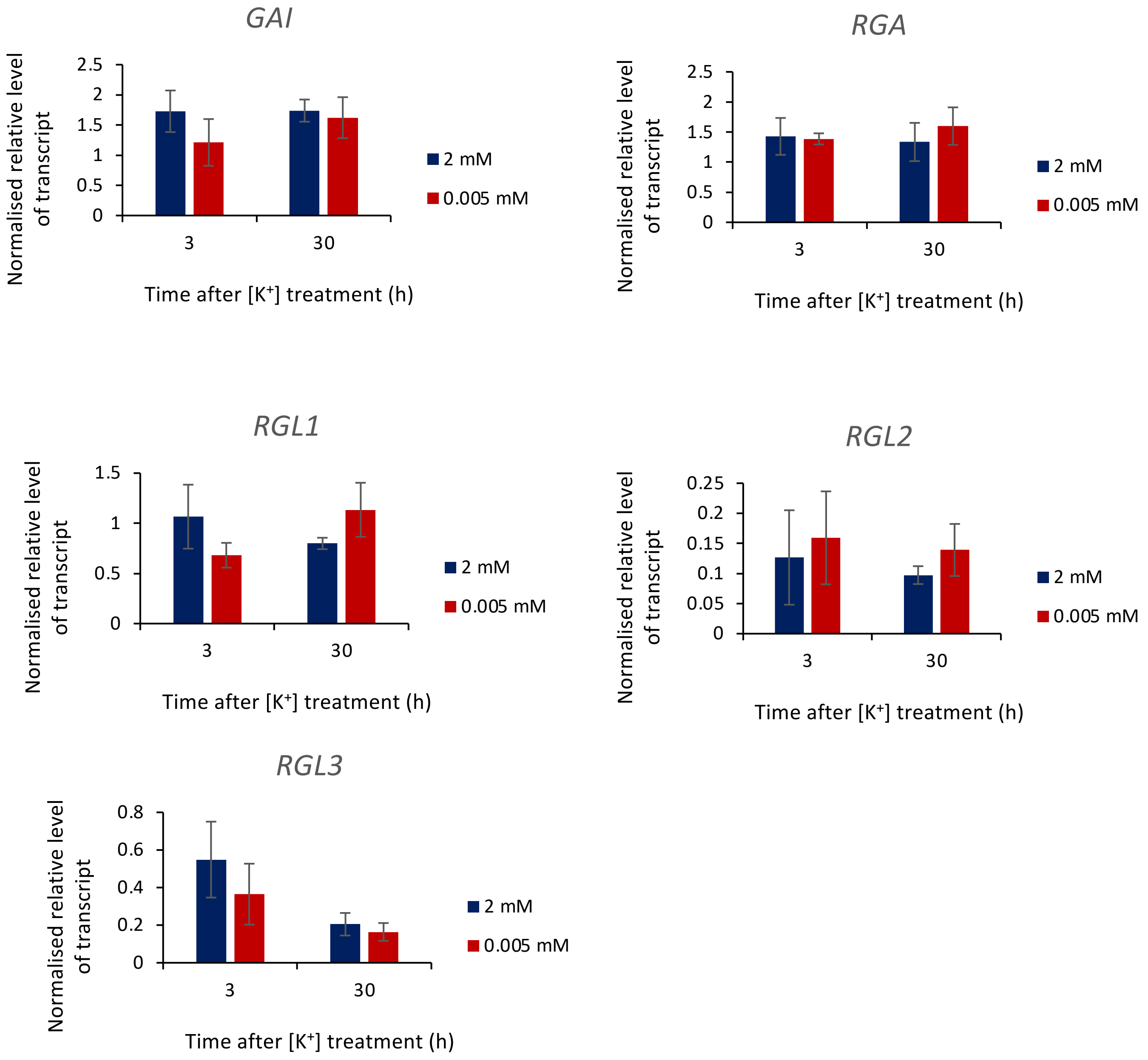

GAI	DELLA	AT1G14920	qRT-PCR	CGTTGAGTCAGTGGAGGAA	TGGCTATGAGCGGTCGTGTGT
RGA	DELLA	AT2G01570	qRT-PCR	AGTTCCGAATAGTCAAGAC	CATCCATTACTCTCCTCCACAC
RGL1	DELLA RGA-LIKE PROTEIN	AT1G66350	qRT-PCR	ACCGGGTAGAGAGGCATGAGACT	CGATTGATTCGCCACGCAGA
RGL2	DELLA RGA-LIKE PROTEIN	AT3G03450	qRT-PCR	CGCTTTACGCTACCGGAGATG	GAGTTTCCACGCCGAGGTTG
RGL3	DELLA RGA-LIKE PROTEIN	AT5G17490	qRT-PCR	GAAGCGCTTCACTATTACTCG	ACTGAGCCAGCGTCTCGTGTC

Supplemental Figure S9. DELLA gene expression following K^+ treatment. Changes in expression of the DELLA genes *GAI, RGA, RGL1*, *RGL2* and *RGL3* after K^+ treatments (2 mM or 0.005 mM [K^+]) at 3 h and 30 h, determined by qRT-PCR and normalised to AT1G13320. Samples taken from seedlings 11 DAG, and values are means ± SE of six biological repeats with three technical repeats.

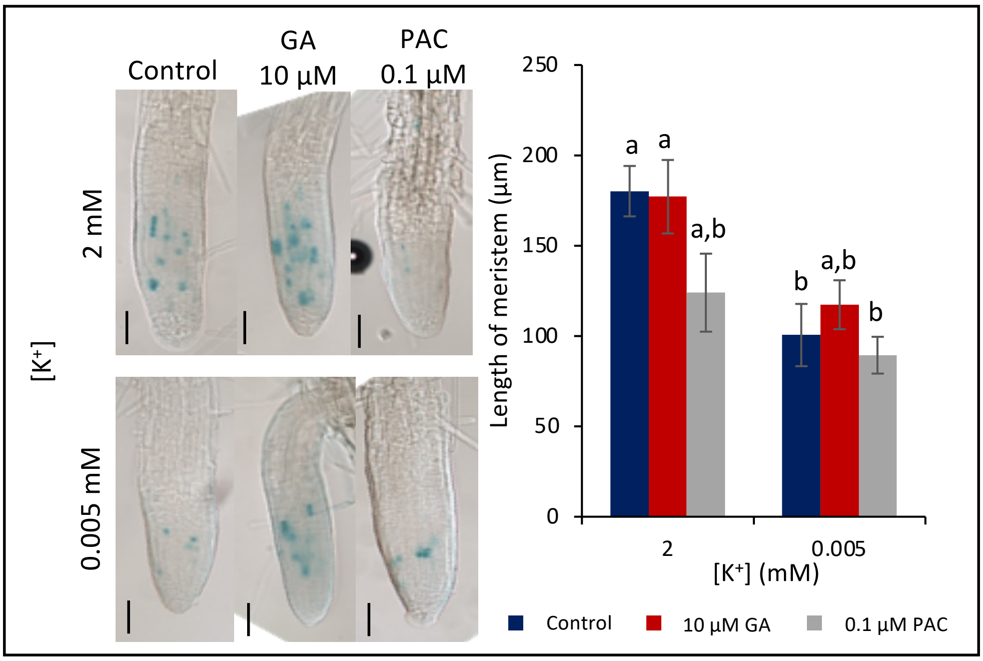

Supplemental Fig. S10. Effect of GA on LR meristem activity under K^+-deprivation. *CYCB1;2:GUS* expression (GUS histochemistry) shows a reduced area of cell division in reduced [K^+]; scale bars = 50 µM; length of meristem measured as the length of root with dividing cells. Lateral roots (LRs) 100 µm-1 mm. Media supplemented with either 10 µM GA or 0.1 µM PAC for 8 d. Analysis carried out on seedlings 12 DAG. Values are mean ± SE for at least 6 individual seedlings per treatment.

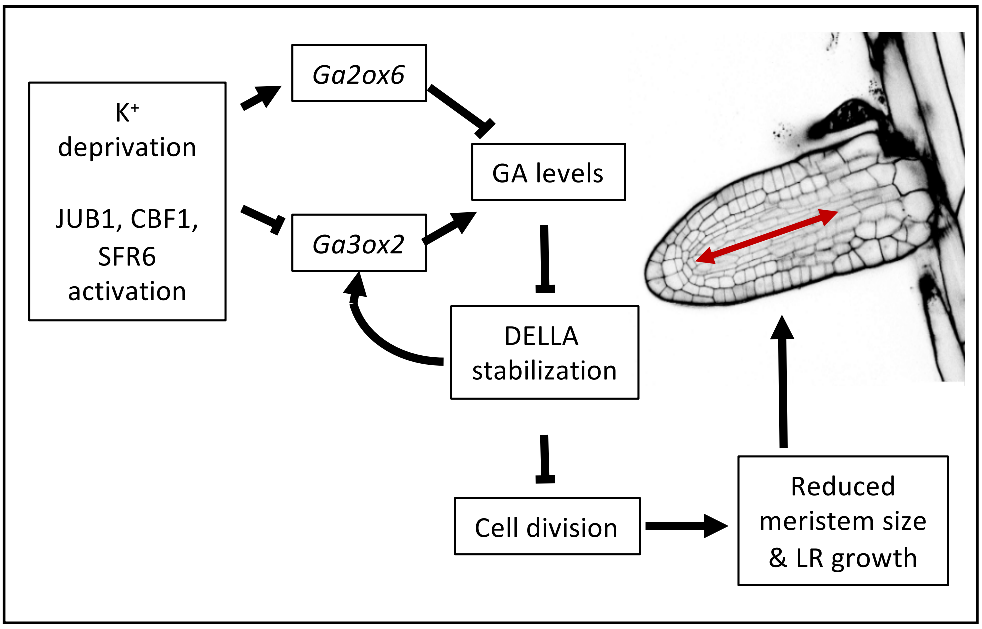

Supplemental Fig. S11. Proposed model for how K^+-deprivation affects LR growth through transient regulation of GA and DELLA levels in Arabidopsis Col-0. Arrows indicate a positive interaction, T-bars indicate inhibition or a negative relationship.

AGI	Gene name	3 h log_2fc	30 h log_2fc
AT1G05575	*AT1G05575*	0.92	0.83
AT1G18570	*MYB51*	0.73	0.52
AT1G21140	*VTL1*		0.65
AT1G26380	*FOX1*		1.32
AT1G27730	*STZ (ZAT10)*	0.83	0.79
AT1G71030	*MYB-like 2*		0.73
AT1G80840	*WRKY40*	1.11	1.29
AT2G26560	*PLP2*		0.65
AT2G38210	*PDX1L4*	0.52	
AT2G43000	*JUB1 (NAC42)*		1.33
AT3G04640	*AT3G04640*	0.50	0.56
AT3G12900	*MJM20.4*		2.00
AT3G14050	*RSH2*		0.61
AT3G15500	*NAC055 (NAC3)*	0.57	0.96
AT3G28580	*AT3G28580*	0.80	0.79
AT3G44260	*CAF1-9*	0.75	0.86
AT3G48850	*MPT2*		0.78
AT3G55980	*AtC3H47*	1.09	0.63
AT4G11280	*ACS6*	0.71	
AT4G17490	*ERF6*	0.93	0.84
AT4G17500	*ERF1A*		0.66
AT4G19690	*IRT1*		0.52
AT4G29780	*AT4G29780*	0.89	0.88
AT4G34710	*ADC2*		0.71
AT4G37370	*CYP81D8*		0.72
AT5G13080	*WRKY75*		0.74
AT5G22250	*CAF1B*		0.63
AT5G27420	*ATL31*	0.70	0.75
AT5G38900	*AT5G38900*		0.63
AT5G47220	*ERF2*		0.98
AT5G47230	*ERF5*	0.52	
AT5G54190	*PORA*		0.67
AT5G54490	*PBP1*	0.87	0.64
AT5G57220	*CYP81F*		0.64

Supplementary Table S2: List of genes both upregulated by K^+ starvation (either after 3 h or 30 h, or both) and also identified by gene ontology (GO) analysis as relating to ethylene signalling. Various GO terms associated with ethylene signalling collated together. Gene names obtained from TAIR.

	Forward Primer	Reverse Primer	Tm (°C)
HK1 *AT1G13320*	TAACGTGGCCAAAATGATGC	GTTCTCCACAACCGCTTGGT	55 59
HAK5	CGAGACGGACAAAGAAGAGGAACC	CACGACCCTTCCCGACCTAATCT	64 64
ERF6	TCGAATCCTCCTCGCGTTACTG	TTCGGTGGTGCGATCTTCAACG	62 62
STZ	TCACAAGGCAAGCCACCGTAAG	TTGTCGCCGACGAGGTTGAATG	62 62
IAA2	CCTCCTACCAAAACTCAAATCGTT	CGTAGCTCACACTGTTGTTGTTCT	59 61
ERF1	GGTATTAGGGTTTGGCTCGG	CCGAAAGCGACTCTTGAACT	58 58
GA2ox6	TGGATCCCAATCCCATCTGACC	TCTCCCATTCGTCAATGCCTGAAG	62 62
GA3ox1	GATCTCCTCTTCTCCGCTGCT	GAGGGATGTTTTCACCGGTG	61 59

Supplemental Table S3: Primer sequences for RT-qPCR.
